# Reprogramming alveolar macrophage responses to TGF-**β** reveals CCR2^+^ monocyte activity that promotes bronchiolitis obliterans syndrome

**DOI:** 10.1172/JCI159229

**Published:** 2022-10-03

**Authors:** Zhiyi Liu, Fuyi Liao, Jihong Zhu, Dequan Zhou, Gyu Seong Heo, Hannah P. Leuhmann, Davide Scozzi, Antanisha Parks, Ramsey Hachem, Derek E. Byers, Laneshia K. Tague, Hrishikesh S. Kulkarni, Marlene Cano, Brian W. Wong, Wenjun Li, Howard J. Huang, Alexander S. Krupnick, Daniel Kreisel, Yongjian Liu, Andrew E. Gelman

**Affiliations:** 1 Department of Surgery,; 2 Department of Radiology, and; 3 Department of Medicine, Washington University School of Medicine, St. Louis, Missouri, USA.; 4 Houston Methodist J.C. Walter Jr. Transplant Center, Houston, Texas, USA.; 5 Department of Surgery, University of Maryland School of Medicine, Baltimore, Maryland, USA.; 6 Department of Pathology & Immunology, Washington University School of Medicine, St. Louis, Missouri, USA.

**Keywords:** Immunology, Inflammation, Adaptive immunity, Monocytes, Organ transplantation

## Abstract

Bronchiolitis obliterans syndrome (BOS) is a major impediment to lung transplant survival and is generally resistant to medical therapy. Extracorporeal photophoresis (ECP) is an immunomodulatory therapy that shows promise in stabilizing BOS patients, but its mechanisms of action are unclear. In a mouse lung transplant model, we show that ECP blunts alloimmune responses and inhibits BOS through lowering airway TGF-β bioavailability without altering its expression. Surprisingly, ECP-treated leukocytes were primarily engulfed by alveolar macrophages (AMs), which were reprogrammed to become less responsive to TGF-β and reduce TGF-β bioavailability through secretion of the TGF-β antagonist decorin. In untreated recipients, high airway TGF-β activity stimulated AMs to express CCL2, leading to CCR2^+^ monocyte-driven BOS development. Moreover, we found TGF-β receptor 2–dependent differentiation of CCR2^+^ monocytes was required for the generation of monocyte-derived AMs, which in turn promoted BOS by expanding tissue-resident memory CD8^+^ T cells that inflicted airway injury through Blimp-1–mediated granzyme B expression. Thus, through studying the effects of ECP, we have identified an AM functional plasticity that controls a TGF-β–dependent network that couples CCR2^+^ monocyte recruitment and differentiation to alloimmunity and BOS.

## Introduction

Bronchiolitis obliterans syndrome (BOS) is the most common form of chronic lung allograft dysfunction (CLAD) and the leading cause of rejection after the first year of transplantation (1). The major pathological hallmark of BOS is the appearance of obliterative bronchiolitis (OB), characterized by peribronchiolar and transluminal fibrotic lesions that restrict airflow (2). OB can also be observed in non–lung transplant settings, such as in patients suffering from graft-versus-host disease or autoimmune diseases (3). The risk of BOS development is linked to nonalloimmune stressors, such as viral or bacterial infection that can cause bronchial injury (2). Club cells play a key role in bronchiolar repair through their capacity to self-renew and differentiate into goblet and ciliated cells (4). Previous work has shown that BOS patients have club cell dysfunction or loss (5). We have recently developed a lung transplant model of BOS that is triggered by the partial depletion of club cells (6). This model utilizes Friend leukemia virus B mouse (FVB) (H-2^q^) donor lungs encoding 3 transgenes (3T-FVB): a reverse tetracycline activator gene driven by the club cell secretory protein (CCSP) promoter, a Cre recombinase gene under the control of the reverse tetracycline activator, and a lox-P–activated diphtheria toxin A gene. When 3T-FVB lungs are transplanted into immunosuppressed MHC-mismatched C57BL/6 (B6; H-2^b^) recipients, club cell depletion after transient doxycycline (DOX) ingestion results in bronchiolar injury and the development of severe OB lesions. Importantly, lymphocyte-mediated immune responses against allo- and autoantigens, known target antigens in BOS subjects, develop in this model (5–7). However, in syngeneic 3T lung transplant recipients, club cell depletion–mediated bronchiolar injury is repaired and fails to produce OB lesions (6).

Extracorporeal photophoresis (ECP) is an autologous cell–based immunotherapy in which apheresed peripheral blood leukocytes are treated with the DNA intercalating compound 8-methoxypsoralen (8-MOP) and ultraviolet A radiation prior to reinfusion. UV-A radiation activates 8-MOP to primarily generate photoadducts with thymine that cause DNA damage and subsequent leukocyte apoptosis (8, 9). ECP has been used for a wide variety of chronic inflammatory disorders and is currently being investigated as treatment for BOS (10–12). Although randomized double-blind trials have yet to be completed, there is accumulating evidence that ECP improves lung function or prevents its decline (13). Additionally, beneficial responses to ECP have been shown to coincide with the reduction of circulating allo- and autoantibodies (14). Previous studies have reported ECP increases TGF-β protein expression, but whether it regulates bioavailability is unclear (15). Although TGF-β is required to maintain tissue homeostasis and helps promote the resolution of inflammation (16), it is also a potent mediator of tissue fibrosis (17). TGF-β is secreted with a bound latent activating peptide and a latent TGF-β–binding protein, which adheres it to the extracellular matrix (18). Following tissue injury, TGF-β can become active through its release from latent complex proteins by a diverse set of factors that include integrin α_v_β_5_, metalloproteases, and cathepsins (19). However, even after becoming active, TGF-β can be reregulated by soluble leucine-rich proteoglycans, such as decorin (DCN), which bind to TGF-β to prevent engagement with its receptor (20).

Alveolar macrophages (AMs) play a critical role in maintaining distal airway homeostasis through promoting host defense and performing surfactant catabolism (21). In quiescent lungs, the AM compartment is nearly entirely composed of self-renewing tissue-resident AMs (TR-AMs) that develop during embryogenesis (22). However, in response to pulmonary injury, TR-AM levels fall, which is coincident with the generation of monocyte-derived AMs (Mo-AMs) (23). In comparison with TR-AMs, the specific requirements for Mo-AM development are less defined. Several reports show that Mo-AMs are derived from bone marrow–derived cells, but have not directly addressed whether this AM subset arises from CCR2^+^ monocytes (24, 25). CCR2 expression on monocytes is critical for trafficking into inflamed lungs in response to the chemokine CCL2 (26, 27). CCR2 expression and Mo-AMs have been shown to drive bleomycin-induced pulmonary fibrosis, raising the possibility that CCR2^+^ monocytes drive pulmonary fibrotic disease through differentiation into Mo-AMs (23, 28). The prevailing view is that AMs are poor antigen-presenting cells that function primarily to enforce airway tolerance (29). However, while controversial, some recent observations have indicated that AMs are capable of promoting the effector activity of tissue-resident memory CD8^+^ T cells (TRM cells ) (30, 31). TRM cells differ from other memory subsets because they do not recirculate and develop in peripheral tissues under the instruction of locally derived cues, such as TGF-β (32, 33). Nevertheless, they share some properties with effector memory cells, such as the expression of the transcription factor *Pdrm1* (Blimp-1), which drives the expression of granzyme b (Gzmb) (34).

Here, by studying the effects of ECP, we uncovered immune mechanisms that promote BOS after lung transplantation. ECP inhibits BOS through reducing AM responses to TGF-β and lowering intragraft TGF-β bioavailability by inducing DCN expression. In untreated recipients, high intragraft TGF-β activity stimulates AM to express CCL2 that in turn drives CCR2^+^ monocyte allograft recruitment and promotes TGF-β receptor 2–dependent CCR2^+^ monocyte differentiation into Mo-AMs. We also observed stable interactions between TRM cells and AMs by intravital 2-photon microscopy and show that Mo-AMs reactivate TRM cells through donor antigen presentation_._ Finally, we demonstrate that Mo-AMs promote BOS through stimulating the expansion of Blimp-1^+^Gzmb^hi^ TRM cells.

## Results

### ECP inhibits BOS and blunts lymphocyte recognition of transplant antigens.

**Figure 1:**
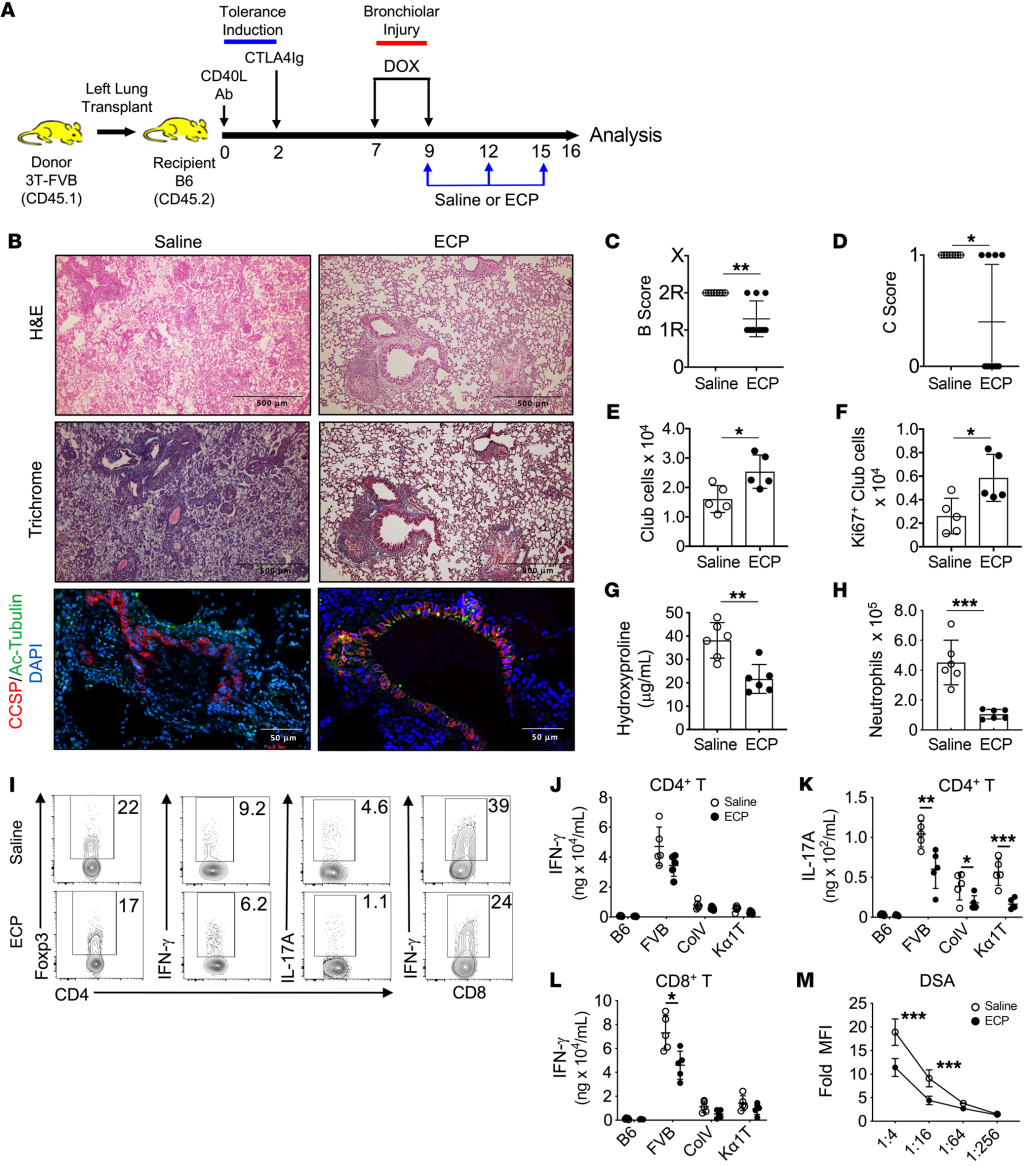
ECP prevents BOS and lymphocyte recognition of transplant antigens. (**A**) 3T-FVB left lungs were transplanted into C57BL/6 (B6) mice and treated with CD40L Abs (POD0) and CTLA4 Ig (POD2) to establish allograft tolerance. Between POD7 and POD9, recipients ingested DOX. They received saline or ECP-treated B6 leukocytes on POD9, POD12, and POD15 and were euthanized on POD16. (**B**) Representative allograft H&E, trichrome, and CCSP/Ac-tubulin Ab staining. Images shown are representative of *n* = 10/group. Allografts scored for airway inflammation (**C**) (B score) and (**D**) the presence (designated 1) or absence (designated 0) of OB lesions (C score) (*n* = 10/group). Intragraft (**E**) total (*n* = 5/group) and (**F**) Ki67^+^ club cell numbers (*n* = 5/group) and (**G**) hydroxyproline content (*n* = 6/group). (**H**) Intragraft neutrophil numbers (*n* = 6/group). (**I**) Representative FACS plots of the intragraft percentage of abundance for indicated T lymphocyte lineages (*n* = 5/group). (**J**–**L**) T cell antigen specificity measured by IFN-γ and IL-17A production following stimulation with splenocytes isolated from B6 (syngeneic antigens), FVB (donor antigens), or B6 mice pulsed with lung self-antigens Col V and Kα1T (*n* = 5/group). (**M**) DSA (IgM) serum reactivity against FVB CD19^+^ cells at indicated dilutions (*n* = 10/group). Assay data shown for **G** and **J**–**L** are representative of at least 2 independent evaluations. Data are represented as mean ± SD. Two-sided Mann Whitney *U* test (**C**–**H** and **J**–**M**). *P < 0.05; **P < 0.01; ***P < 0.001.

To analyze the immunoregulatory effects of ECP, we utilized mouse donor lungs (3T-FVB) that, when transplanted into immunosuppressed B6 recipients, develop BOS following DOX-mediated ingestion to induce diphtheria toxin expression in club cells (6). Following DOX ingestion, lung recipients received i.v. infusions of ECP-treated B6 leukocytes at 3-day intervals and allografts were analyzed for histological appearance and lymphocyte activation on postoperative day 16 (POD16) (Figure 1A). Allografts were assessed for airway inflammation (B score), where 0 = none, 1R= low grade, 2R = high grade, and X = ungradable and the presence (designated 1) or absence (designated 0) of OB (C score) in accordance with the 2007 revision of the International Society for Heart and Lung Transplantation working formulation for the diagnosis of lung rejection (35). In contrast with saline vehicle–treated mice, recipients that received ECP had significantly less peribronchiolar inflammation, were largely devoid of OB lesions, and were significantly able to regenerate club cells (Figure 1, B–F). ECP treatment also reduced intragraft hydroxyproline content and neutrophilia (Figure 1, G and H). Analysis of allograft infiltrate revealed lower levels of IL-17A^+^CD4^+^ and IFN-γ^+^CD8^+^ T cells (Figure 1I and Supplemental Figure 1A; supplemental material available online with this article; https://doi.org/10.1172/JCI159229DS1). We next assessed the effects of ECP on transplant-antigen recognition by lymphocytes (Figure 1, J–M, and Supplemental Figure 1B). When compared with those of saline-treated recipients, CD4^+^ and CD8^+^ T cells from ECP-treated recipients had reduced CD4^+^ T cell–mediated IL-17 production and less CD8^+^ T cell–mediated IFN-γ production following restimulation with donor antigens. Subjects that develop BOS have been reported to have lymphocytes that recognize the lung self-antigens collagen V (Col V) and k-α tubulin (Kα1T) (7, 14). ECP-treated allograft resident CD4^+^ T cells, when challenged with Col V or Kα1T peptides, expressed less IL-17A when compared with cells from saline-treated recipients. Finally, and in line with previous clinical observations (14), ECP reduced the serum levels of donor-specific Abs (DSAs). These results demonstrate that ECP inhibits transplant antigen-specific responses and reduces BOS severity.

### ECP reprograms AM to inhibit intragraft TGF-β bioavailability.

**Figure 2:**
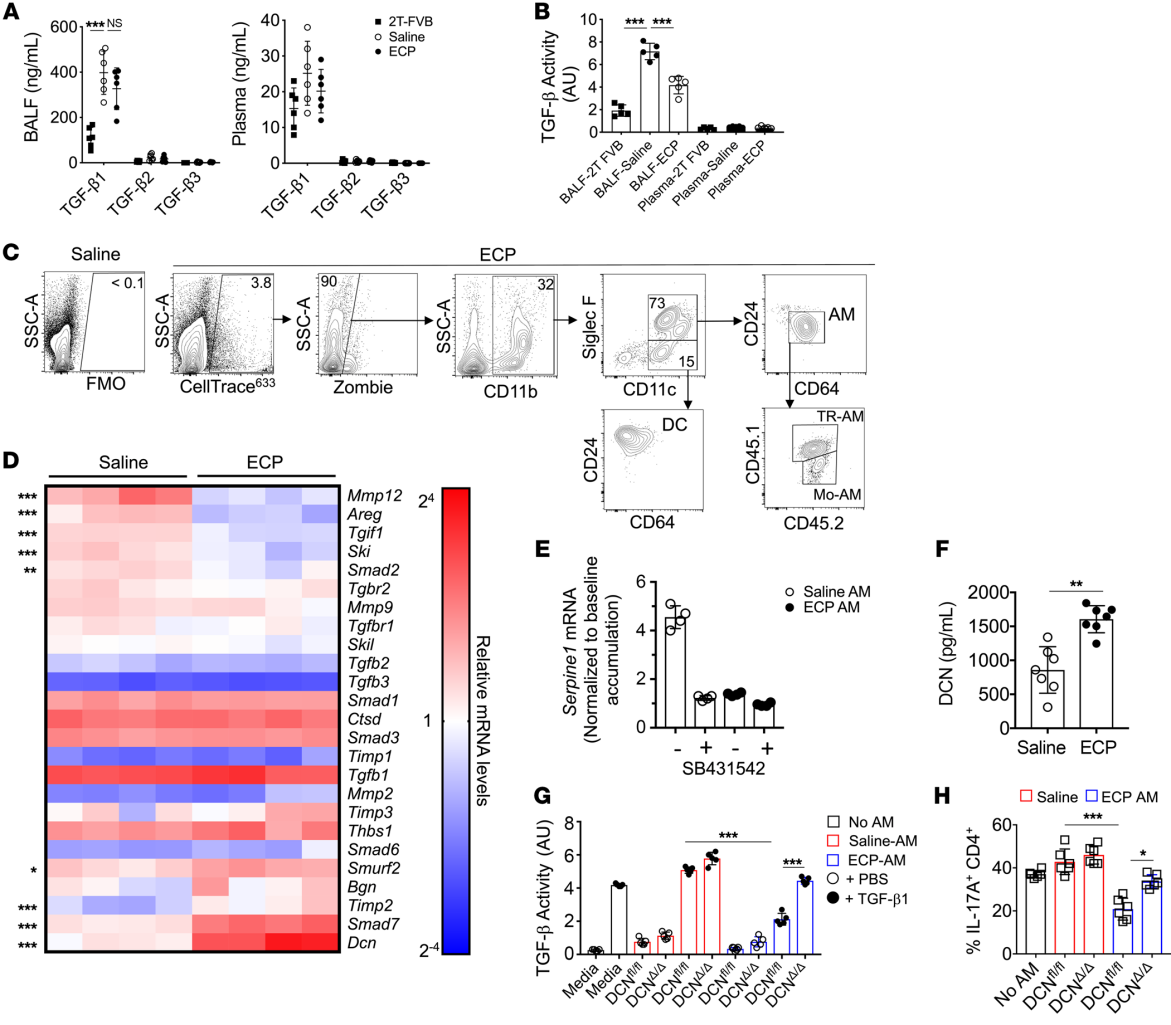
ECP reprograms AMs to antagonize TGF-β bioavailability. POD16 2T-FVB and 3T-FVB allograft (**A**) BALF and plasma analyzed for TGF-β isoform protein content by ELISA (*n* = 6/group) or (**B**) activity with a HEK293 SMAD 2/3 luciferase reporter cell line (*n* = 5/group). AU, arbitrary luciferase units. Data shown for **A** and **B** are representative results from 2 experiments. (**C**) CellTrace^633^-labeled ECP-treated leukocytes injected into 3T-FVB allograft and analyzed for uptake by intragraft CD11b^+^ phagocytes. Data shown are representative results from 4 experiments. (**D**) Heatmap of saline- and ECP-treated POD16 3T-FVB allografts, AM transcript levels of TGF-β signaling, and fibrosis-related gene targets normalized to the macrophage housekeeping gene *Stx5a*. (*n* = 4/group) (**E**) Fold accumulation of TGF-β–induced AM *Serpine1* mRNA accumulation in the presence or absence of 10 μM SB43152 or vehicle (DMSO) (*n* = 4/group). Data shown are normalized to baseline levels (non–TGF-β–treated DMSO-pretreated controls). (**F**) Saline- and ECP-treated AMs were cultured overnight and analyzed by ELISA for DCN secretion (*n* = 7/group). (**G**) TGF-β activity measurements of enriched supernatants from saline- or ECP-treated DCN^Δ/Δ^ and DCN^fl/fl^ AMs cultured with or without 10 ng/ml TGF-β1 (*n* = 5/group). Data shown in **F** and **G** are representative results from 2 experiments. (**H**) Naive B6 CD4^+^ T cells were stimulated with plate-bound CD3ε and CD28 Abs in the presence or absence of indicated AM-conditioned supernatants added at a 1:1 v/v ratio to Th17 polarization medium that contained 10 ng/ml TGF-β1 (*n* = 5/group). Intracellular IL-17A expression was assessed 4 days later. Data are represented as mean ± SD. One-way ANOVA with Dunnett’s multiple-comparison test (**A**, **B**, **G**, and **H**); 2-sided Mann-Whitney *U* test (**D** and **F**). *P < 0.05; **P < 0.01; ***P < 0.001.

Given reports that ECP stimulates TGF-β production (15), we measured protein levels of all 3 TGF-β isoforms in the bronchoalveolar lavage fluid (BALF) and the peripheral serum of saline- and ECP-treated 3T-FVB as well as 2T-FVB lung recipients, which maintained established tolerance despite DOX ingestion, as they did not undergo bronchiolar injury due to the lack of the *lox-P* activated diphtheria toxin A gene (6) (Figure 2A). Relative to those in 2T-FVB lung recipients, BALF TGF-β1 levels were markedly elevated in saline- and ECP-treated 3T-FVB recipients. However, BALF TGF-β1 accumulation was not significantly different between saline- and ECP-treated 3T-FVB recipients. Additionally, BALF and circulating TGF-β2 and TGF-β3 levels were either undetectable or were expressed in very modest quantities in all lung recipients. Because TGF-β bioavailability is highly regulated (19), we next assessed the ability of lung recipient BALF and peripheral serum to induce TGF-β–receptor signaling using a SMAD2/3 reporter cell line (Figure 2B). BALF from ECP-treated recipients had substantially less TGF-β activity when compared with that of saline-treated 3T-FVB hosts. In contrast, no significant differences in TGF-β activity were detected in the peripheral blood of saline- and ECP-treated 3T-FVB and 2T-FVB lung recipients. In light of recent observations that immunoregulatory circuits act locally within lung transplants to control tolerance (36), we next analyzed ECP-treated leukocyte trafficking to 3T-FVB allografts just prior to the induction of BOS pathogenesis. ECP-treated cells were labeled with a fluorescent dye and assessed for engulfment by 3T-FVB allograft CD11b^+^ phagocytes 2 hours after i.v. injection (Figure 2C and Supplemental Figure 2). ECP-treated leukocytes were predominantly engulfed by TR-AMs and Mo-AMs, which could be identified by CD45.1 and CD45.2 expression in the donor lung and recipient, respectively.

Given that AM uptake of ECP-treated leukocytes was linked to lower airway TGF-β bioavailability, we next analyzed 3T-FVB allograft AM transcript levels of 25 genes reported to control TGF-β responses and activation in lung macrophages (25, 37, 38) (Figure 2D). Nine transcripts were found to be differentially regulated by ECP. For example, several genes that inhibit TGF-β signaling, *Dcn*, *Smad7*, and *Smurf2,* were significantly upregulated in ECP-treated AMs (39). Conversely, factors that promote latent TGF-β activation, such as *Mmp13* and *Areg* (40), were downregulated by ECP. Interestingly, genes that regulate TGF-β signaling, such as *Tgif1* and *Ski*, were also downregulated in ECP-treated AMs, suggesting a compensatory response due to a lack of homeostatic TGF-β receptor signaling (25). To further confirm these observations, we analyzed TGF-β1–mediated responses of 2 known TGF-β expression targets, *Serpine1* (41) (Figure 2E) and the TGF-β–activating integrin α_v_β_5_ (42) (Supplemental Figure 3), in saline- and ECP-treated AMs. Unlike in ECP-treated AMs, TGF-β1 induced *Serpine1* mRNA accumulation and α_v_β_5_ protein upregulation in saline-treated AMs. To determine whether these reductions were TGF-β signaling dependent, ECP- and saline-treated AMs were also preincubated with the TGF-β receptor inhibitor SB431542 (43) prior to stimulation with TGF-β1. SB431542 addition to saline-treated AMs inhibited TGF-β1–mediated *Serpine1* and α_v_β_5_ expression to levels nearly comparable to those of TGF-β1–stimulated ECP-treated AMs.

Since DCN was the most differentially regulated transcript in our analysis, we measured its secretion (Figure 2F). DCN secretion was significantly higher from ECP-treated AMs when compared with saline-treated AMs. Additionally, immunofluorescence staining of ECP-treated lung allograft tissue showed enhanced DCN expression within CD64^+^ macrophages, many of which were located in or around alveoli (Supplemental Figure 4). We further sought to determine whether ECP-treated AMs regulate TGF-β activity in a DCN-dependent manner. For this purpose, we generated *Lyz^Cre/+^Dcn^fl/fl^* (DCN^Δ/Δ^) mice and tested the ability of conditioned supernatants from ECP-treated DCN^Δ/Δ^ and WT control *Dcn^fl/fl^* (DCN^fl/fl^) AMs to stimulate TGF-β signaling activity (Figure 2G). Supernatants from ECP-treated DCN^fl/fl^ AMs sharply reduced TGF-β activity when compared with ECP-treated DCN^Δ/Δ^ AMs or saline-treated DCN^fl/fl^ AMs. Notably, alterations in TGF-β activity were most apparent when TGF-β1 was added to cultures, indicating ECP-treated AMs primarily target TGF-β activity generated by exogeneous sources. TGF-β drives Th17 generation from naive CD4^+^ T cells (44) and also promotes Th17 lineage stability (45). Given that ECP treatment reduces intragraft IL-17A^+^CD4^+^ T cell accumulation, we analyzed the effects of saline- and ECP-treated AM-conditioned supernatants on Th17 cell development (Figure 2H and Supplemental Figure 5). Differentiation of naive CD4^+^ T cells into IL-17A^+^CD4^+^ T cells was impeded by ECP-treated DCN^fl/fl^ AMs when compared with ECP-treated DCN^Δ/Δ^ AMs or saline-treated AMs irrespective of DCN expression. Collectively, these data show that ECP induces AMs to become less responsive to TGF-β signals and also reduces local TGF-β bioavailability.

### ECP-mediated inhibition of BOS is dependent on AM DCN expression.

**Figure 3:**
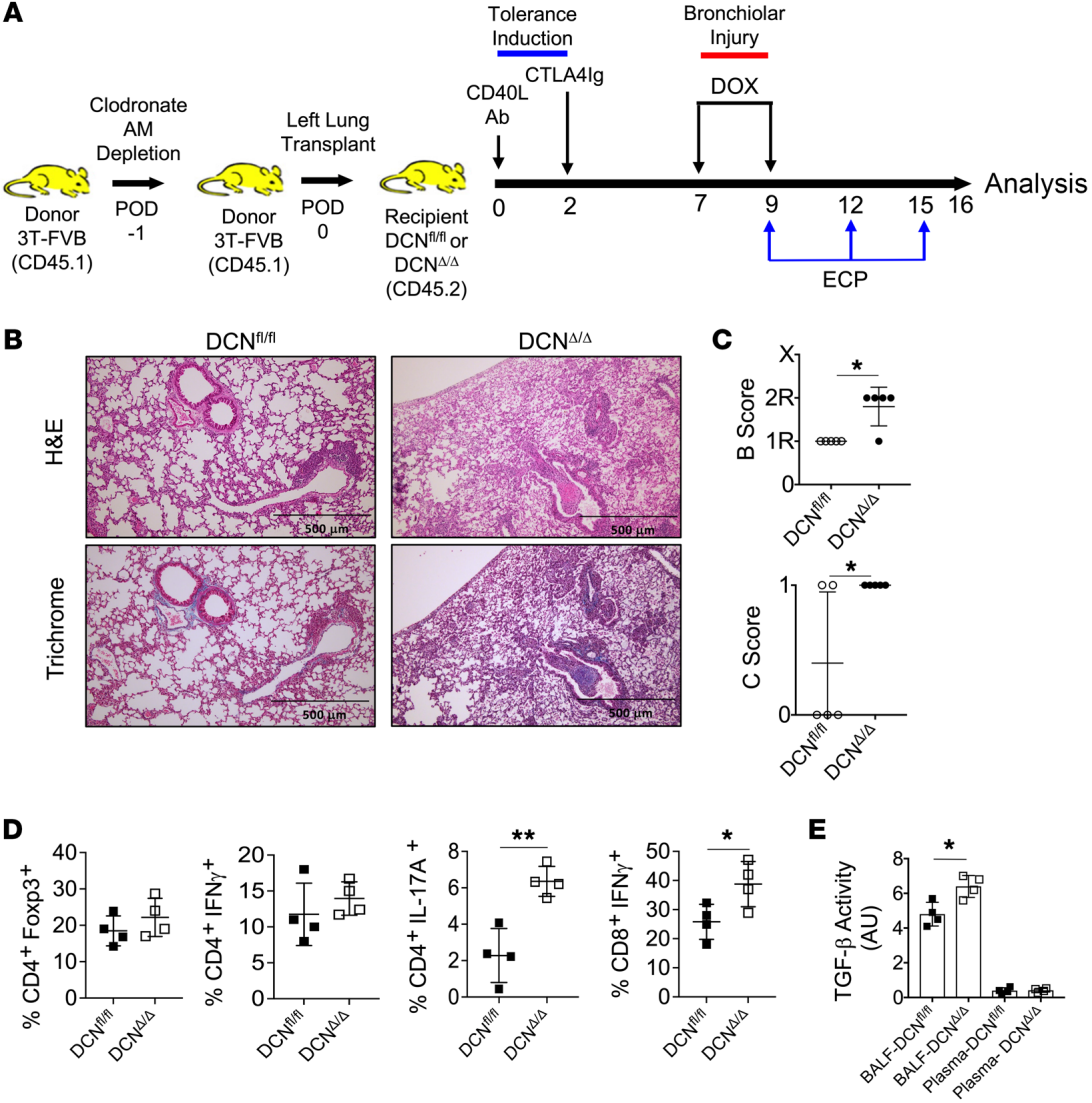
AM DCN expression is required for ECP-mediated inhibition of BOS. (**A**) One day prior to transplantation (POD1) into DCN^Δ/Δ^ and DCN^fl/fl^ recipients, 3T-FVB lung donors were treated with intratracheal clodronate liposomes (100 μL) to deplete airway AMs. ECP treatment was conducted between POD9 and POD15, and on POD16, intragraft inflammation was evaluated and is shown by a (**B**) representative image of H&E and trichrome staining (*n* = 5/group) and graphs showing (**C**) airway inflammation and lesion grading (*n* = 5/group), (**D**) intragraft T cell activation (*n* = 4/group), and (**E**) BALF and circulating plasma TGF-β activity (*n* = 4/group). Data shown in **E** are representative results from 2 experiments. Data are represented as mean ± SD. Two-sided Mann-Whitney *U* test (**C**–**E**). *P < 0.05; **P < 0.01.

**Figure 4:**
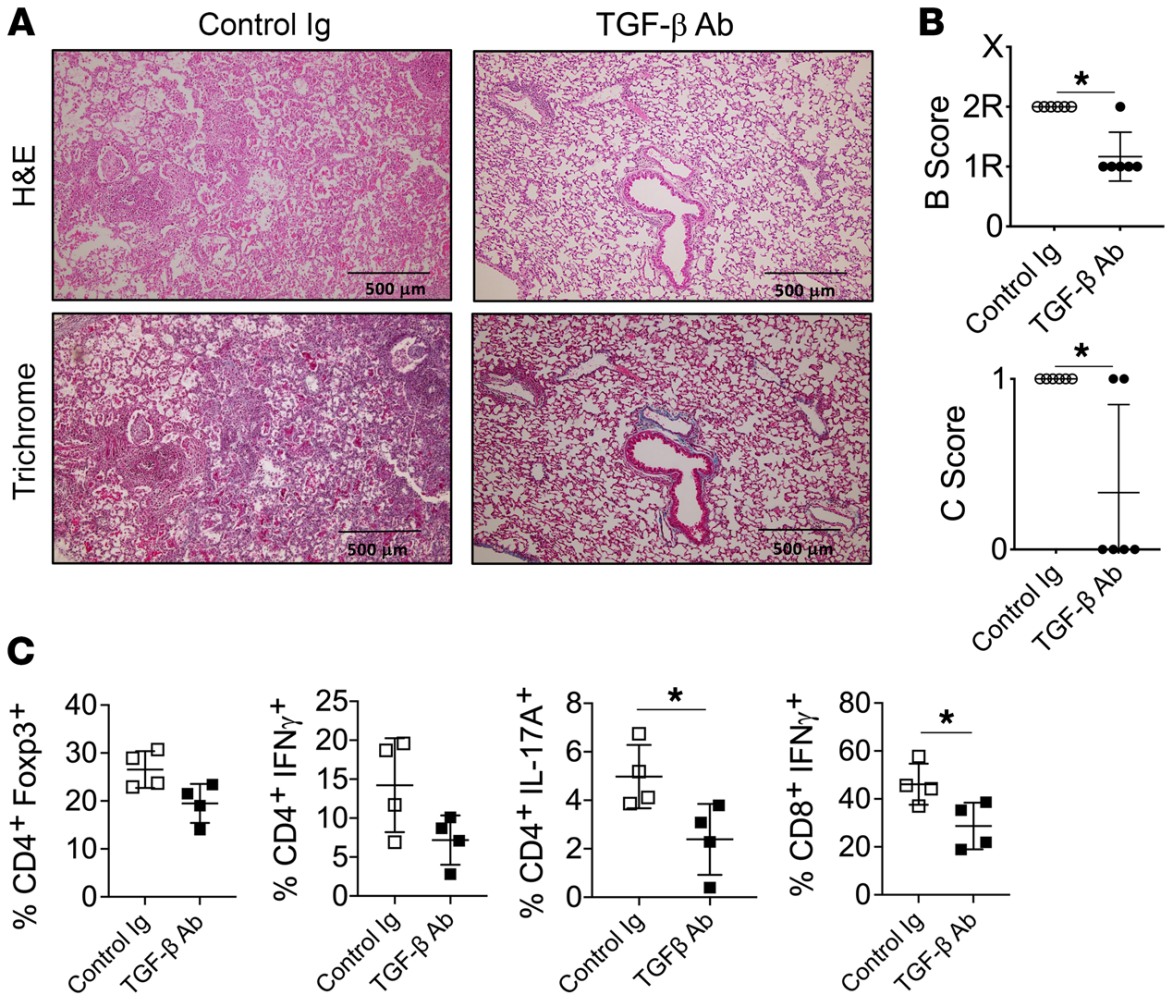
TGF-β blockade prevents intragraft IFN-γ^+^CD8^+^ T cell accumulation and BOS. B6 recipients of 3T-FVB allografts received intratracheal mouse IgG or TGF-β Abs (75 μg/100 μL PBS) on POD7 and on POD16 were analyzed for intragraft inflammation as shown by (**A**) a representative image of H&E and trichrome staining (*n* = 6/group), (**B**) airway inflammation and OB lesion scoring (*n* = 6/group), and (**C**) intragraft T cell activation (*n* = 4/group). Data are represented as mean ± SD. Two-sided Mann-Whitney *U* test (**B** and **C**). *P < 0.05.

To determine whether AM DCN expression is required for ECP-mediated attenuation of BOS, we first replaced donor allograft AMs with DCN^Δ/Δ^ or DCN^fl/fl^ AMs by administering clodronate liposomes into the trachea of donor lung 3T-FVB mice 1 day prior to transplantation into respective DCN^Δ/Δ^ or DCN^fl/fl^ recipients (Figure 3A). Importantly, clodronate treatment led to an approximately 95% reconstitution of the AM compartment with recipient-derived AMs, but did not prevent the induction of immunosuppression-mediated acceptance or spontaneously induce BOS lesions (Supplemental Figure 6, A–E). However, following bronchiolar injury, ECP was ineffective at attenuating BOS and failed to reduce IL-17A^+^CD4^+^ or IFN-γ^+^CD8^+^ T cell intragraft accumulation in DCN^Δ/Δ^ recipients (Figure 3, B–E). In contrast, ECP-treated WT DCN^fl/fl^ recipients were protected from BOS and had lower BALF TGF-β activity when compared with ECP-treated DCN^Δ/Δ^ recipients. Because DCN is reported to interact with other growth factors that regulate inflammation (46), it remained possible that our observed effects on TGF-β activity were unrelated to inhibiting BOS development. To see whether this is true, we tested the effects of TGF-β Ab blockade on BOS development (Figure 4, A–C). TGF-β neutralizing Abs were administered intratracheally into B6 recipients of 3T-FVB lungs and induced to undergo BOS pathogenesis. T cell activation and OB lesion generation were inhibited in a manner comparable to that of ECP treatment. Overall, these data indicate that AM-mediated regulation of TGF-β bioavailability controls BOS pathogenesis.

### Infiltrating CCR2^+^ monocytes promote BOS.

**Figure 5:**
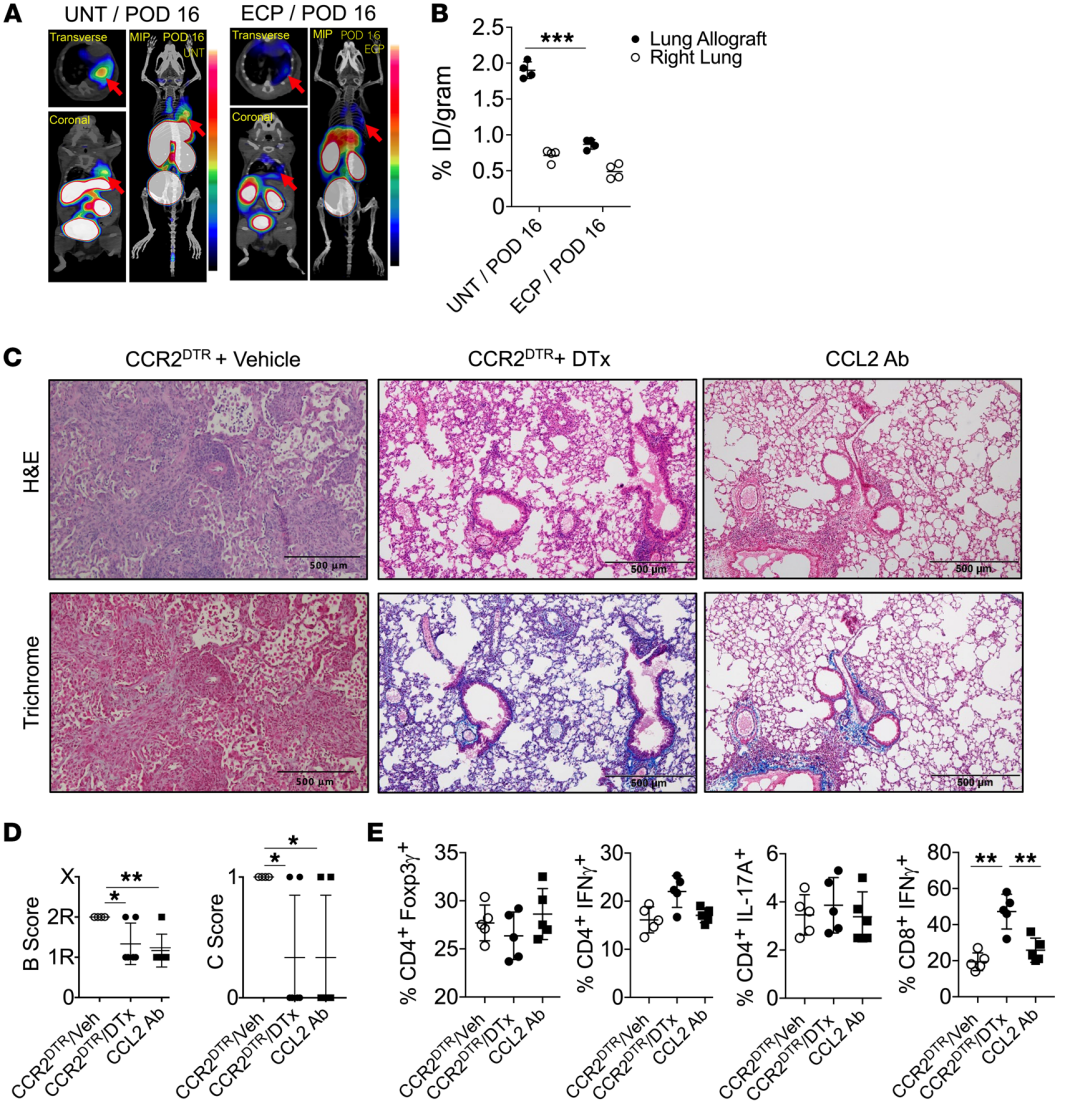
Targeting CCR2 expression inhibits BOS. (**A**) Dynamic ^64^Cu-DOTA-ECL1i PET/CT image scans of untreated and ECP-treated 3T-FVB allografts (red arrows) with (**B**) right native lung and allograft probe uptake quantitation shown as percentage of injected dose per gram (%/ID/gram) of tissue (*n* = 4/group). Images shown are representative results from 4 scans. (**C**) 3T-FVB allografts of CCR2^DTR^ recipients that received 10 ng/g i.v. of diphtheria toxin on POD6 and POD11 and B6 recipients of 3T-FVB allografts that received 200 μg i.v. of CCL2-neutralizing Abs on POD6, POD9, and POD12. Both recipients were euthanized on POD16 and assessed for intragraft inflammation by (**C**) representative H&E and trichrome staining (*n* = 5/group), (**D**) airway inflammation and lesion grading (*n* = 5/group), and (**E**) intragraft T cell activation (*n* = 5/group). Data are represented as mean ± SD. One-way ANOVA with Dunnett’s multiple-comparison test (**B**, **D**, and **E**). *P < 0.05; **P < 0.01; ***P < 0.001.

Given previous reports that recipient CCR2 deficiency prevents fibrosis in mouse nonvascularized tracheal allografts (47), we next set out to assess ECP-mediated changes in CCR2 expression within lung allografts. To this end, we imaged ECP-treated lung recipients using a PET-purposed radiotracer, ^64^Cu-DOTA-ECL1i, which specifically recognizes the extracellular loop number 1 of CCR2 and is under current clinical evaluation for noninvasive diagnosis of idiopathic pulmonary fibrosis (48). When compared with untreated 3T-FVB allografts that develop BOS, allografts of recipients treated with ECP showed a sharp decrease in CCR2 activity (Figure 5, A and B). We next determined whether CCR2^+^ monocytes are required for BOS development. 3T-FVB lungs were transplanted into CCR2^DTR^ recipients, which express the diphtheria toxin receptor under the control of the CCR2 promoter (49) and were depleted of CCR2^+^ monocytes following diphtheria toxin treatment. We also inhibited the activity of CCL2 by injecting CCL2-neutralizing Abs into B6 recipients of 3T-FVB allografts (Figure 5, C–E, and Supplemental Figure 7, A and B). CCR2^+^ monocyte depletion or CCL2 Ab blockade reduced the development of severe OB lesions. Additionally, we observed a sharp reduction in intragraft IFN-γ^+^CD8^+^ T cells when compared with cells under control conditions. Collectively, our data indicate that CCR2^+^ monocytes promote BOS.

### TGF-β stimulates AM CCL2 expression to promote Mo-AM allograft accumulation.

**Figure 6:**
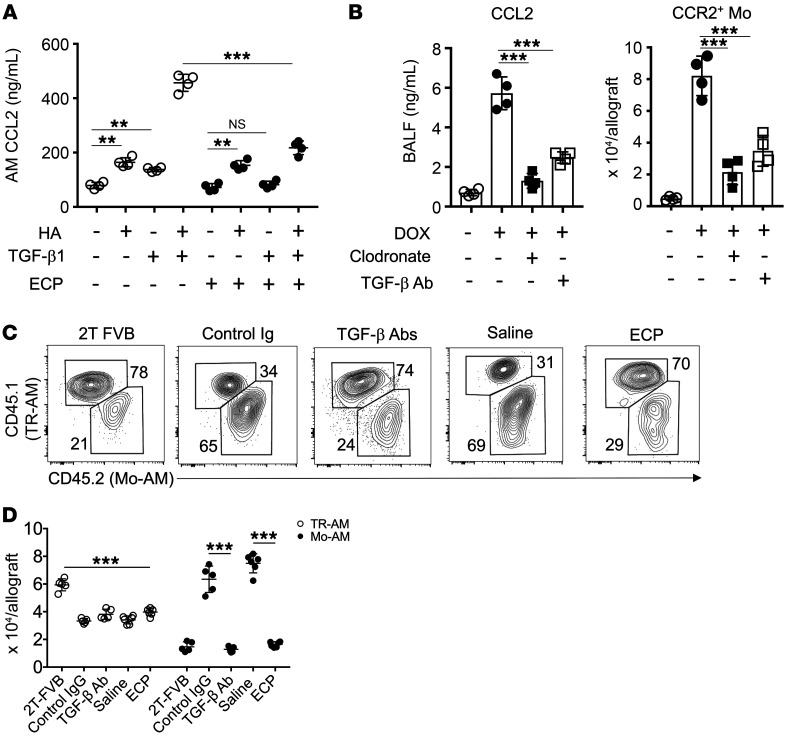
A TGF-β-AM-CCL2 expression circuit promotes Mo-AM allograft accumulation. (**A**) Untreated and ECP-treated AMs were stimulated with 10 ng/ml TGF-β1 and/or 1 μg/ml HA for 18 hours and assessed for CCL2 expression by ELISA. Data shown are representative results of 3 experiments with *n* = 4/stimulation. (**B**) POD6 3T-FVB allograft recipients were treated with intratracheal TGF-β Abs (75 μg/100 μL PBS) or clodronate liposomes (100 μL), induced to undergo bronchiolar injury (DOX) on POD7, and assessed for BALF CCL2 expression and CCR2^+^ monocyte recruitment on POD8 (*n* = 4/treatment). 2T-FVB and 3T-FVB allograft recipients underwent indicated treatments and were quantitated for Mo-AMs and TR-AMs, as shown by a representative contour plot of (**C**) percentage of abundance (*n* ≥ 5/group) and (**D**) cell counts (*n* ≥ 5/group). Data are represented as mean ± SD. One-way ANOVA with Dunnett’s multiple-comparison test (**A**, **B**, and **D**). **P < 0.01; ***P < 0.001.

AMs are reported to produce CCL2 after lung transplantation (27). TGF-β targets activation of AP-1 and EGR1, transcription factors that promote CCL2 gene transcription (50, 51). Noting these previous observations, we stimulated saline- and ECP-treated AMs with TGF-β1 and measured CCL2 production (Figure 6A). TGF-β1 induced CCL2 expression in saline-, but not ECP-treated, AMs. Hyaluronic acid (HA), a damage-associated molecular pattern molecule that we have shown accumulates in lung transplants with BOS (52), has also been demonstrated to promote CCL2 expression in a mouse AM cell line (53). HA was found to accumulate in 3T-FVB allograft airways (Supplemental Figure 8A), and its addition to TGF-β1–stimulated cultures induced a synergistic increase in CCL2 expression in saline-treated AMs relative to those treated with HA stimulation alone. In ECP-treated AMs, CCL2 expression mediated by TGF-β ΗΑ costimulation was comparable to that with HA stimulation alone, indicating a lack of a synergistic response in these cells. We next asked whether AM-mediated CCL2 production during BOS pathogenesis induces CCR2^+^ monocyte allograft infiltration (Figure 6B). 3T-FVB allografts were depleted of AMs or treated with anti–TGF-β Abs prior to bronchiolar injury and assessed for airway CCL2 production and numbers of recruited CCR2^+^ monocytes. Although we could detect some CCL2 production in allografts prior to bronchiolar injury, levels rose approximately 6-fold following bronchiolar injury. In contrast, CCL2 levels were substantially reduced by either AM depletion or TGF-β Ab blockade, with the remaining CCL2 expression possibly emanating from airway epithelial cells (54). Moreover, either treatment potently blunted CCR2^+^ monocyte allograft accumulation.

With regard to previous observations that Mo-AMs drive pulmonary fibrogenesis (23), we quantified Mo-AMs and TR-AMs in 3T-FVB allograft recipients treated with saline, ECP, control Ig, or TGF-β–neutralizing Abs (Figure 6, C and D). Relative to 2T-FVB allograft recipients with established tolerance, we observed a nearly uniform reduction in TR-AMs in 3T-FVB allografts irrespective of treatment. In contrast, Mo-AM levels were affected by treatments that target TGF-β bioavailability. In control Ig- or saline-treated lung recipients, Mo-AMs were approximately 2-fold more abundant than TR-AMs and were approximately 4 times more numerous when compared with Mo-AMs in TGF-β Ab– or ECP-treated allografts. Moreover, we also observed a sharp reduction in Mo-AMs in diptheria toxin–treated CCR2^DTR^ and CCL2 Ab–treated recipients, indicative of a CCR2^+^ monocyte origin (Supplemental Figure 8B). Collectively, these data indicate that TGF-β induces AMs to express CCL2, which in turn promotes the allograft recruitment of CCR2^+^ monocytes and Mo-AMs.

### TGF-β receptor–mediated CCR2^+^ monocyte differentiation into Mo-AM leads to BOS.

**Figure 7:**
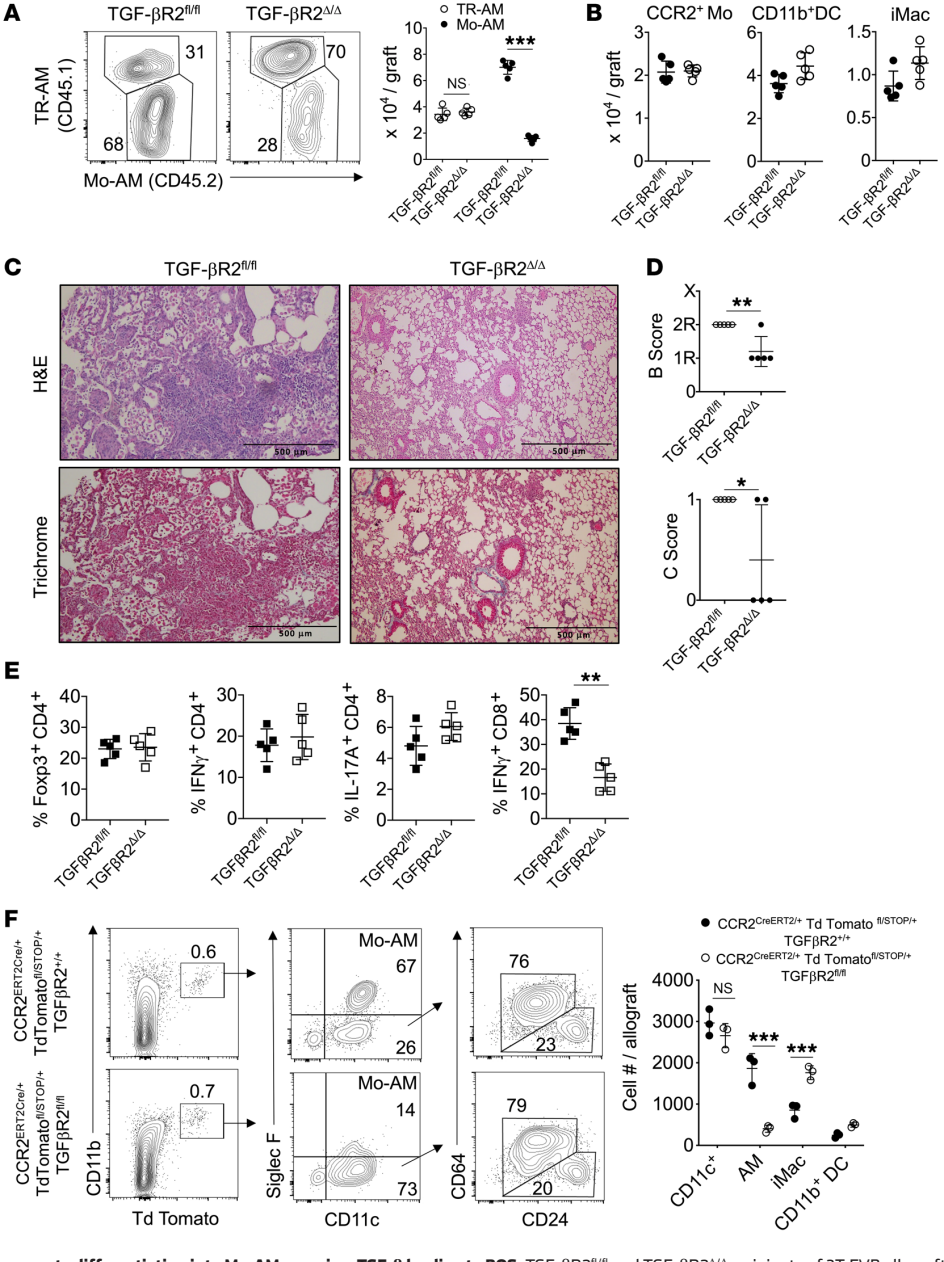
CCR2^+^ monocyte differentiation into Mo-AMs requires TGF-β leading to BOS. TGF-βR2^fl/fl^ and TGF-βR2^Δ/Δ^ recipients of 3T-FVB allografts received tamoxifen i.p. every other day for 10 days, rested for 5 days, and then ingested DOX for 2 days. Eight days later, allograft recipients were analyzed for intragraft inflammation (**A**), as shown by representative FACS plots of the relative percentage of abundance of Mo-AMs and TR-AMs with cell counts (*n* = 5/group), (**B**) CCR2^+^ monocytes (Mo), CD11c^+^ DCs, and iMac cell counts (*n* = 5/group), (**C**) representative H&E and trichrome staining (*n* = 5/group), (**D**) airway inflammation and lesion grading (*n* =5/group), and (**E**) intragraft T cell activation (*n* = 5/group). (**F**) 3 × 10^6^ FACS-purified CCR2^+^ bone marrow monocytes were isolated from indicated Td Tomato reporter mice that received tamoxifen as in **A** and were injected into POD7 3T-FVB recipients undergoing BOS pathogenesis. On POD16, allograft tissues were quantified for Td Tomato^+^ Mo-AMs, CD11b^+^ DCs, and iMacs, as shown by representative FACS plots and cell counts. FACS plots shown are a representative result of 3 experiments. Data are represented as mean ± SD. Two-sided Mann-Whitney *U* test (**A**, **B**, and **D**–**F**). *P < 0.05; **P < 0.01; ***P < 0.001.

The correlation between CCR2^+^ monocyte and Mo-AM allograft accumulation during BOS pathogenesis raised the possibility that CCR2^+^ monocytes differentiate into Mo-AMs. Interestingly, previous work has shown that TGF-β promotes AM development from bone marrow–derived cells (25). To determine whether TGF-β drives CCR2^+^ monocyte differentiation into Mo-AMs, we generated tamoxifen-inducible *Cc2r^CreERT2/+^* *Tgfbr2^fl/fl^* (TGF-βR2^Δ/Δ^) mice. Tamoxifen treatment of TGF-βR2^Δ/Δ^ CCR2^+^ monocytes reduced *Tgfbr2* mRNA by nearly 80% relative to that in *Tgfbr2^fl/fl^* (TGF-βR2^fl/fl^) controls and inhibited TGF-β1–mediated generation of 2 transcripts required for AM development, *Pparg* and *Car4* (55) (Supplemental Figure 9, A and B). TGF-βR2^Δ/Δ^ recipients of 3T-FVB lungs were comparatively poor at inducing Mo-AM accumulation when compared with WT TGF-βR2^fl/fl^ recipients (Figure 7A). Notably, the sharp reduction in Mo-AM graft accumulation in TGF-βR2^Δ/Δ^ recipients was not due to a defect in CCR2^+^ monocyte recruitment following bronchiolar injury (Figure 7B). Moreover, TGF-βR2^Δ/Δ^ lung recipients were significantly protected from BOS, which was associated with a reduction in intragraft IFN-γ^+^CD8^+^ T cells (Figure 7, C–E). As the reduction in BOS could be explained by the inability of CCR2^+^ monocytes to differentiate into other CD11c^+^ descendants, such as interstitial macrophages (iMacs) or CD11b^+^ DCs, we created reporter *Ccr2^CreERT2/+^ Tgfbr2^fl/fl^* *TdTomato^fl/STOP/+^* and *Ccr2^CreERT2/+^ Tgfbr2^+/+^* *TdTomato^fl/STOP/+^* mice to conduct fate-mapping studies. Following tamoxifen treatment, CCR2^+^ monocytes were adoptively transferred into 3T-FVB lung transplant recipients undergoing BOS pathogenesis (Figure 7F). Irrespective of TGF-βR2 deletion, we detected similar numbers of CD11c^+^ descendants within allografts. However, TGF-βR2–deficient CCR2^+^ monocytes were profoundly deficient at generating Mo-AMs. In contrast, iMacs and CD11b^+^ DCs developed independently of TGF-βR2. Collectively, these data indicate that intrinsic CCR2^+^ monocyte TGF-β signaling is required for Mo-AM development, but not for the generation of other CD11c^+^-derived lineages.

### Mo-AMs promote TRM cell activation and expansion.

**Figure 8:**
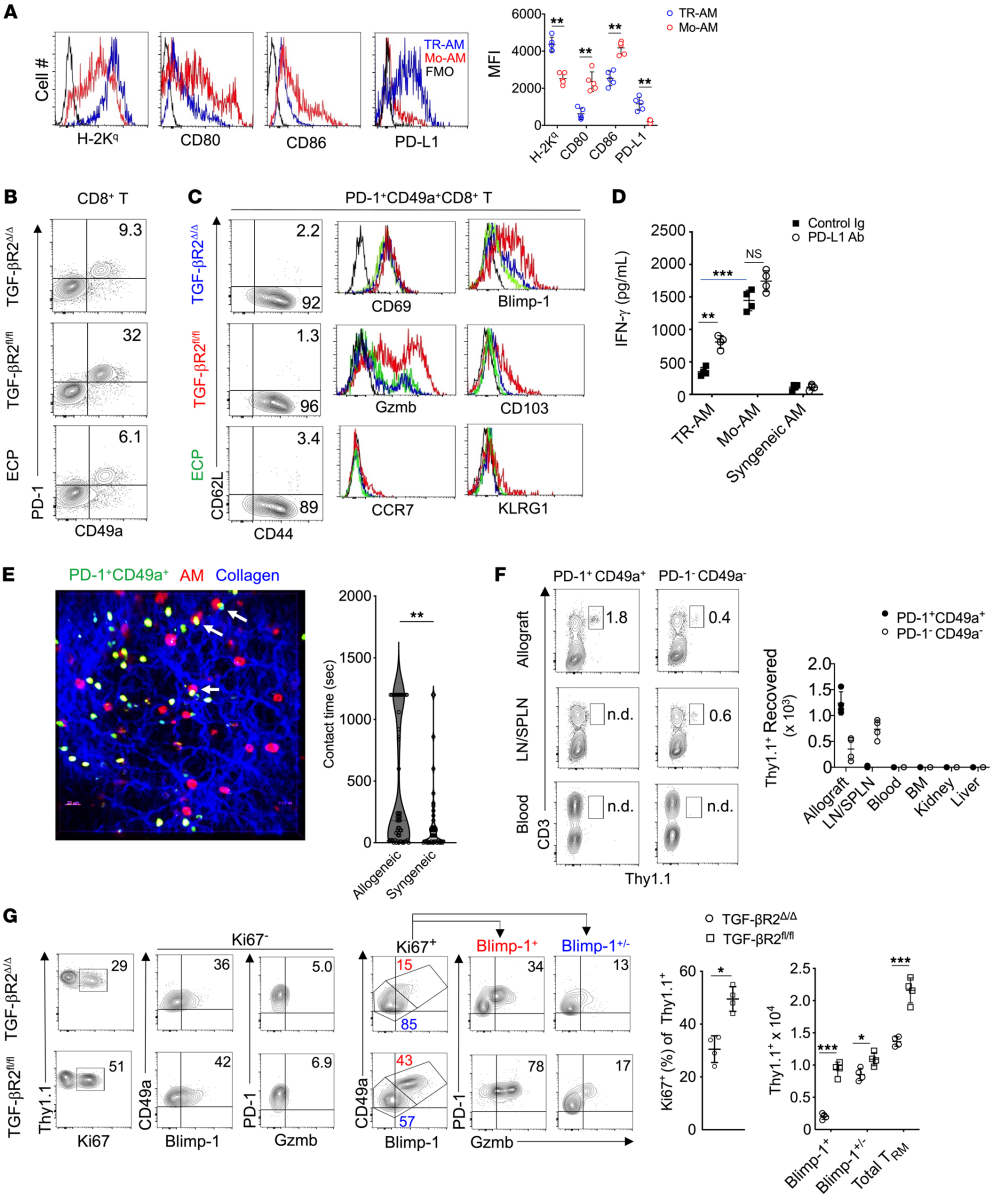
Mo-AM generation promotes TRM cell activation and expansion. (**A**) A representative FACS plot set from 4 transplants where MFI is shown for TR-AM and Mo-AM MHC I H-2K^q^, CD80, CD86, and PD-L1 expression levels. FMO, fluorescence-minus-one control. (**B** and **C**) Representative FACS plots and histograms for *n* = 4/group for the expression of TRM cell markers with FMO (black lines). (**D**) FACS-sorted 3T-FVB TR-AMs, Mo-AMs, and B6 AMs were cultured with FACS-sorted 3T-FVB allograft PD-1^+^CD49a^+^CD8^+^ T cells with 10 μg/ml control rat Ig or PD-L1–neutralizing Abs and then assessed for IFN-γ production by ELISA 72 hours later. Data shown are representative results from 2 experiments. (**E**) FACS-sorted, CFSE-labeled 3T-FVB allograft PD-1^+^CD49a^+^CD8^+^ T cells (green) were intratracheally administered to FVB (allogeneic) or B6 (syngeneic) lung transplants of B6 recipients. Eighteen hours later, transplants were imaged by 2-photon intravital microscopy immediately following the administration of Siglec F Abs to identify AMs (red). Representative intravital image from 1 of 4 FVB-transplanted lung studies. Arrows denote long-lasting contacts between TRM cells and AMs. Right panel shows violin plot of individual AM-TRM cell contact times from pooled data from 4 FVB (allogeneic) or B6 (syngeneic) transplanted lungs. (**F**) 2T-FVB allografts of B6 Thy1.1^+^ recipients were FACS-sorted for PD-1^+^CD49a^+^ and PD-1^–^CD49a^–^ CD8^+^ T cells and intratracheally delivered into B6 Thy1.2^+^ recipients of 2T-FVB allografts and euthanized 1 month later. Shown are representative FACS plot results of Thy1.1^+^ cell percentage of abundance and cell count for indicated tissues (*n* = 4 per adoptive transfer). (**G**) 2T-FVB allograft FACS-sorted Thy1.1 PD-1^+^CD49a^+^CD8^+^ T cells were intratracheally administered into tamoxifen-treated TGF-βR2^fl/fl^ and TGF-βR2^Δ/Δ^ recipients of 3T-FVB allografts 3 days after DOX ingestion. Seventy-two hours later, recipients were euthanized. Data shown are representative FACS plots from *n* = 4/group for allograft percentage of abundance and cell counts. Data are represented as mean ± SD. Two-sided Mann-Whitney *U* test (**A** and **G**); 1-way ANOVA with Dunnett’s multiple-comparison test (**D**). *P < 0.05; **P < 0.01; ***P < 0.001.

Recent work has shown that human lung–resident macrophages colocalize to TRM cells (56), and several other reports have demonstrated TRM cell maintenance and activation patterns that are associated with lung transplant outcomes (57, 58). We have previously demonstrated that donor antigen–primed effector CD8^+^ T cells prevent club cell proliferation and that CD8^+^ T cells are critical mediators of BOS development (6). In light of the correlation between Mo-AMs and IFN-γ^+^CD8^+^ T cell accumulation in allografts with BOS, we next set out to analyze the expression of surface molecules on Mo-AMs that control effector CD8^+^ T cell activation (Figure 8A). Consistent with our previous observations that lung allograft–infiltrating CCR2^+^ monocyte–derived cells express donor-derived MHC molecules, examination of Mo-AMs from 3T-FVB transplants revealed the acquisition of the donor-derived MHC class I molecule H-2K^q^ (26). However, unlike TR-AMs, Mo-AMs lacked expression of the checkpoint inhibitory molecule PD-L1 and had higher levels of costimulatory ligands CD80 and CD86. We next analyzed patterns of PD-1 expression in the CD8^+^ T cell compartment of 3T-FVB lung transplants of TGF-βR2^Δ/Δ^, TGF-βR2^fl/fl^, and ECP-treated recipients (Figure 8B and Supplemental Figure 10A). In TGF-βR2^fl/fl^ recipients, approximately one-third of allograft-resident CD8^+^ T cells coexpressed PD-1 and the integrin CD49a (59). In contrast, allografts of TGF-βR2^Δ/Δ^ and ECP-treated recipients had substantially fewer PD-1^+^CD49a^+^CD8^+^ T cells. Despite differences in abundance, the PD-1^+^CD49a^+^CD8^+^ T cell compartment in all 3 allograft recipients exhibited a similar TRM cell phenotype (60) (Figure 8C). Nearly all PD-1^+^CD49a^+^CD8^+^ T cells were CD44^+^, but lacked expression of CD62L, CCR7, and the killer-like receptor G1 (KLRG1), indicating they were not central memory or short-lived effector cells (61, 62). Additionally, PD-1^+^CD49a^+^CD8^+^ T cells did not express additional checkpoint inhibitory molecules, such as TIM-3 and LAG-3, indicating they were not exhausted memory cells (63) (Supplemental Figure 10B). Moreover, we detected similar TRM cell phenotypes and abundance in 2T-FVB allografts, demonstrating that these cells exist in accepted lung transplants prior to the development of BOS (Supplemental Figure 10C). However, PD-1^+^CD49a^+^CD8^+^ T cells in allografts of TGF-βR2^fl/fl^ recipients expressed moderate levels of the TRM cell marker CD103 and high levels of Gzmb and Blimp-1.

Viral peptide- and alloantigen-specific TRM cells can become reactivated upon cognate antigen encounter (30, 64). We next isolated PD-1^+^CD49a^+^CD8^+^ T cells from 3T-FVB allografts with BOS and measured IFN-γ expression in response to stimulation with 3T-FVB allograft–derived Mo-AMs and TR-AMs (Figure 8D). TR-AMs were poor at eliciting IFN-γ production when compared with Mo-AMs. However, the addition of anti–PD-L1 Abs to TR-AM, but not Mo-AM, cocultures significantly increased IFN-γ responses. Although these data indicated that TR-AM and Mo-AM differentially regulate TRM cell activation responses through PD-L1 expression, it remained unclear whether these cells directly interact with AMs within lung transplants. To answer this question, we utilized intravital 2-photon microscopy to assess contact times between PD-1^+^CD49a^+^CD8^+^ T cells and AMs (Figure 8E and Supplemental Videos 1 and 2). CFSE-labeled PD-1^+^CD49a^+^CD8^+^ T cells isolated from 3T-FVB allografts were intratracheally delivered into FVB lung (allogeneic) or control syngeneic B6 lung recipients. One day later, lung recipients also received Siglec-F fluorescently labeled Abs to identify AMs. Relative to syngeneic B6 lung transplants, significantly prolonged interactions were observed between AMs and PD-1^+^CD49a^+^CD8^+^ T cells in FVB allografts, which is indicative of donor-antigen recognition (65, 66). A canonical property of TRM cells is their inability to exit from barrier organs to recirculate in the periphery (64). To determine whether this was the case for PD-1^+^CD49a^+^CD8^+^ T cells, we isolated Thy1.1^+^PD-1^+^CD49a^+^CD8^+^ T cells from 2T-FVB allografts of Thy1.1^+^ B6 recipients and intratracheally delivered these cells into 2T-FVB allografts of Thy1.2^+^ B6 recipients (Figure 8F). One month later, we could detect Thy1.1^+^ cells in lung allograft tissue, but not in secondary lymphoid organs, peripheral blood, bone marrow, liver, or kidney. In contrast, 2T-FVB allograft–derived Thy1.1^+^PD-1^–^CD49a^–^CD8^+^ T cells were detected in secondary lymphoid organs, indicating that intratracheally administered CD8^+^ T cells can exit lung allografts. Therefore, lung allograft PD-1^+^CD49a^+^CD8^+^ T cells are phenotypically and functionally consistent with TRM cells, and herein we will refer to these cells as TRM cells.

Recent work in models of cutaneous viral infection has indicated that TRM cells expand from a local preexisting population of TRM cells, but whether this is true for pulmonary TRM cells is less understood (67). Because we noted that TRM cells are more abundant in BOS compared with accepted allografts, we asked whether Mo-AM generation during BOS development drives the local expansion of these cells from preexisting intragraft pools. Therefore, we adoptively transferred Thy1.1^+^ TRM cells from accepted 2T-FVB allografts into 3T-FVB allograft airways of TGF-βR2^fl/fl^ and TGF-βR2^Δ/Δ^ Thy1.2^+^ recipients following tamoxifen treatment and bronchiolar injury (Figure 8G). Five days later, intragraft Thy1.1^+^ TRM cells were analyzed for proliferation and accumulation. In allografts of TGF-βR2^fl/fl^ recipients, Thy1.1^+^ TRM cells proliferated and accumulated at higher levels when compared with TGF-βR2^Δ/Δ^ recipients. Analysis of the proliferating Thy1.1^+^ TRM cell compartment of TGF-βR2^fl/fl^ allograft recipients revealed high numbers of PD-1^+^Blimp-1^+^CD8^+^ T cells that expressed elevated Gzmb, which was similar in phenotype to the native TRM cell phenotype detected in these allografts. Additionally, both allografts contained proliferating PD-1^+^Blimp-1^+/–^Gzmb^+/–^ CD8^+^ T cells, a phenotype that resembled the native TRM cell allograft compartment observed in TGF-βR2^Δ/Δ^ and ECP-treated recipients. Finally, we detected clusters of Gzmb^+^CD49a^+^CD8^+^ cells in explanted lung transplant tissue from BOS patients that were not present in stable recipients that did not have evidence of rejection (Supplemental Figure 11). Collectively, our observations indicate that Mo-AM generation during BOS pathogenesis drives the activation and expansion of TRM cells.

### TRM cell Gzmb expression induces airway epithelial apoptosis and promotes BOS.

**Figure 9:**
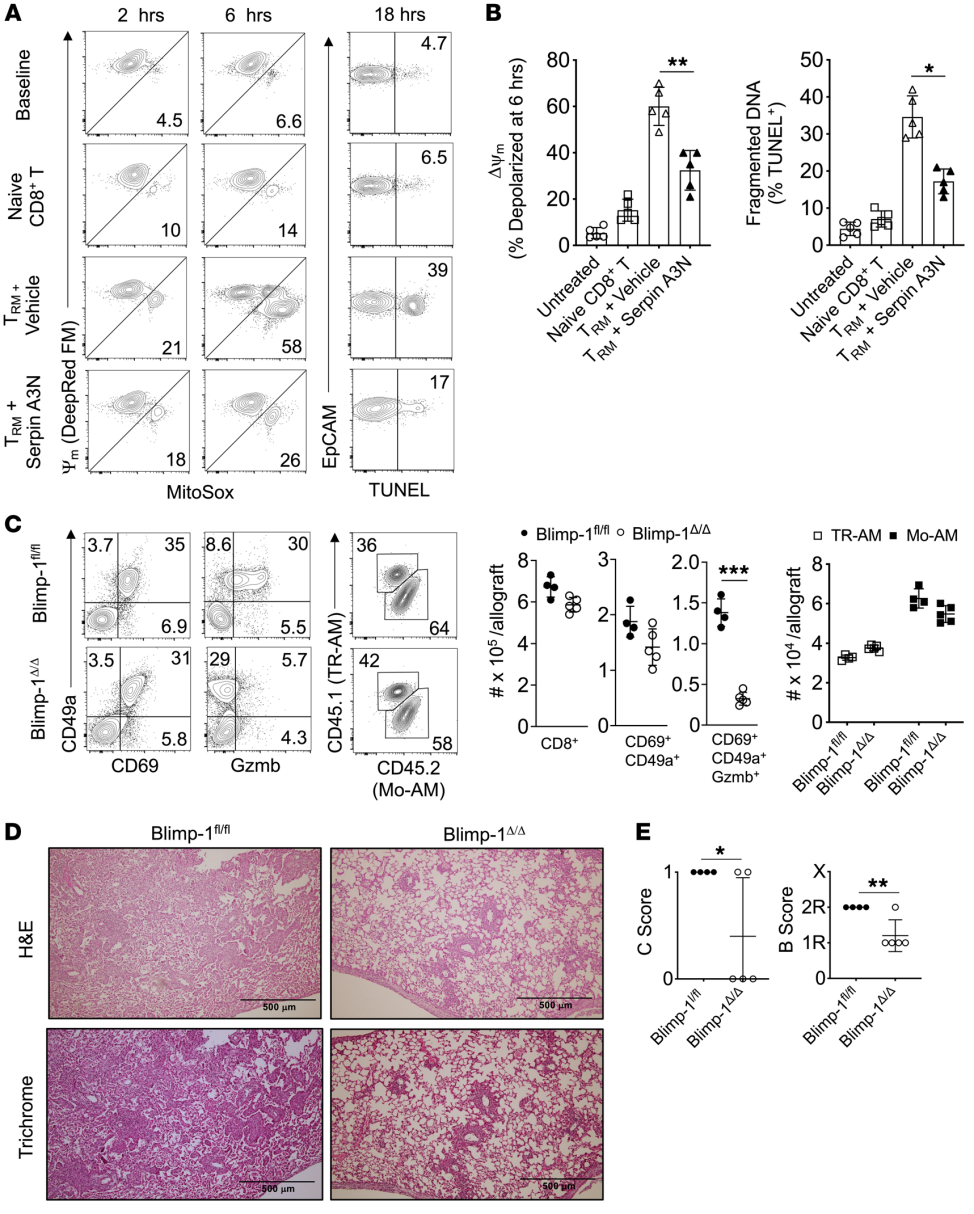
Gzmb^+^ TRM cells promote airway epithelial cell apoptosis and BOS through Blimp-1. FVB lung epithelial cells were cocultured in a 1:2 EpCAM^+^ cell–to–CD8^+^ T cell ratio for up to 18 hours with or without Serpin A3N pretreatment (25 nM) and assessed for mitochondrial membrane potential (MitoTracker Deep Red FM), mitochondrial superoxide production (MitoSOX), and DNA fragmentation (TUNEL). Data are shown as (**A**) a representative FACS plot result from 5 experiments and (**B**) 6-hour epithelial cell mitochondrial depolarization and TUNEL activity (*n* = 5/condition). Blimp-1^fl/fl^ and Blimp-1^Δ/Δ^ recipients of 3T-FVB allografts were analyzed for intragraft inflammation as shown by (**C**) representative FACS plot data of TRM cell markers, Gzmb expression, and AM abundance, with cell counts *n* ≥ 4/group. (**D**) Representative H&E and trichrome staining results for *n* ≥ 4/group and (**E**) airway inflammation and lesion grading (*n* ≥ 4 /group). Data are represented as mean ± SD. One-way ANOVA with Dunnett’s multiple-comparison test (**B**); 2-sided Mann-Whitney U test (**C** and **E**).*P < 0.05; **P < 0.01.

Gzmb induces mitochondrial stress leading to apoptosis and has been reported to be elevated in the BALF of BOS subjects (68, 69). The finding of high Gzmb expression in TRM cells from allografts with BOS indicated the potential to promote airway epithelial cell cytotoxicity. We isolated TRM cells from 3T-FVB allografts with BOS for coculture with lung epithelial cells from FVB mice and measured changes in mitochondrial membrane potential, mitochondrial ROS production, and DNA fragmentation in the presence or absence of the Gzmb inhibitor Serpin A3N (70) (Figure 9, A and B). TRM cells induced rapid mitochondrial stress, as evidenced by mitochondrial membrane depolarization and elevated superoxide production. Additionally, DNA fragmentation, an indicator of late-stage apoptosis, was more than 5-fold greater relative to that in control naive B6 CD8^+^ T cell cocultures. In contrast, TRM cell–induced mitochondrial stress and DNA fragmentation could be inhibited by pretreatment with Serpin A3N. These data indicate that BOS allograft–derived TRM cells induce airway injury through Gzmb expression.

Blimp-1 has been shown to drive Gzmb expression in mouse TRM cells (34). The observation of coexpression of Blimp-1 with high amounts of Gzmb in TRM cells from allografts with BOS raised the possibility that it plays a role in promoting rejection. We next used *CD8a*^Cre^ *Pdrm1*^fl/fl^ (Blimp-1^Δ/Δ^) mice as recipients for 3T-FVB lungs and assessed intragraft inflammation and BOS severity. When compared with that in *Pdrm1*^fl/fl^ (Blimp-1^fl/fl^) recipients, we observed similar numbers of total intragraft CD8^+^ T cells and TRM cells, with little effect on CD69 and CD49a expression (Figure 9C and Supplemental Figure 12). However, Blimp-1^Δ/Δ^ TRM cells were largely devoid of Gzmb expression and their allografts were markedly protected from severe BOS despite maintaining high numbers of Mo-AMs (Figure 9, D and E).

## Discussion

Devising therapies to prevent or treat chronic rejection is one of the major challenges in the transplantation field. Advances in BOS treatment have been largely hampered by our incomplete understanding of the contributory immune mechanisms that drive airway damage. Given some encouraging reports of ECP treatment in BOS patients, we reasoned that we could gain insight into the underlying mechanisms of BOS through modeling this therapy in mouse orthotopic lung transplants. Consistent with observations in ECP-treated subjects with BOS (14), we noted a sharp reduction in lung transplant antigen-specific responses in our model. We also made the clinically relevant observation that ECP ameliorates OB lesion severity (13).

Most of the investigative focus into TGF-β–mediated fibrogenesis has led to the elucidation of mechanisms that control extracellular matrix remodeling, epithelial-to-mesenchymal transition, and fibrogenesis. Although these pathways are involved in BOS pathogenesis (71), the additional requirement for leukocyte-dependent recognition of alloantigens mechanistically distinguishes BOS from other pulmonary fibrotic diseases. We were initially surprised by reports of ECP’s effectiveness in BOS patients, given that previous studies have demonstrated that ECP stimulates TGF-β protein expression along with the expansion of Foxp3^+^CD4^+^ T cells (15). However, we did not find that ECP increased TGF-β protein expression or expansion of Foxp3^+^CD4^+^ T cells, which suggests that different mechanisms drive ECP-mediated immunoregulatory effects in lung transplant recipients. Surprisingly, we discovered that ECP prevents BOS through inhibiting lung airway TGF-β activity by inducing AM DCN expression. In contrast, peripheral TGF-β activity was not affected by ECP. These observations suggest that ECP promotes lung transplant survival through targeting local alloimmune responses. Although we cannot exclude the possibility that ECP’s beneficial effects are also dependent on inducing immunoregulatory responses within secondary lymphoid organs, our previous work has established that lung allograft rejection is unimpeded in splenectomized alymphoplastic mice (66). In this respect, lung transplants are unique when compared with other vascularized allografts that require secondary lymphoid organs for rejection (72), and this potentially explains why ECP-mediated immunoregulatory effects in mouse cardiac allograft recipients act through peripheral mechanisms (73). Moreover, we have also previously demonstrated, that once immunosuppression-induced lung allograft acceptance is established, retransplantation of lung allografts into genetically identical nonimmunosuppressed recipients does not abrogate tolerance (36, 74, 75), suggesting that immunoregulatory circuits that are critical for long-term survival reside within pulmonary tissues. Collectively, these series of observations support our strategy of evaluating local immune cell activity to probe ECP mechanisms.

Noninvasive approaches to detecting early BOS development have yet to be developed due to a lack of knowledge of the underlying immunological mechanisms that lead to OB. Using CCR2^+^ probe–based micro-PET imaging, we detected a large increase in CCR2^+^ intragraft activity in lung recipients with BOS that was largely reversed by ECP treatment. Whether all intragraft activity was from CCR2^+^ monocytes is less clear, as DCs, NK cells, and lymphocytes can express CCR2 to varying degrees (76). However, we detected large numbers of CCR2^+^ monocytes in lung allografts with BOS, suggesting that the bulk of PET activity is due to the infiltration of CCR2^+^ monocytes.

A recent study employing single-cell RNA-Seq revealed that the majority of AMs in human lung allografts are derived from the recipient (77). These observations led us to consider the relevance of Mo-AM accumulation during BOS development. Notably, we observed a high Mo-AM–to–TR-AM ratio in BOS allografts that was driven by a combination of CCR2^+^ monocyte differentiation and a reduction in TR-AM numbers following bronchiolar injury. The reasons for the loss of TR-AMs are not clear, but it was not preventable by ECP or anti–TGF-β Ab treatment. Therefore, their loss could be potentially explained by their programmed cell death following airway inflammation (78). We recognized that targeting CCR2^+^ monocyte–mediated Mo-AM depletion could also result in the defective generation of other CCR2^+^ monocyte–derived cells that may contribute to BOS, such as iMacs and CD11b^+^ DCs (26). Following total body irradiation, lysosomal M–mediated expression of TGF-βR2 has been reported as required for AM reconstitution, raising the possibility that CCR2^+^ monocytes give rise to Mo-AMs (25). We conducted monocyte fate studies with reporter mice in which TGF-βR2 deletion and Td tomato expression were both under the inducible control of CCR2 cre recombinase. In recognition of the leakiness of the Td-tomato “flox on” constructs (79), we studied the differentiation of FACS-purified CCR2^+^ monocytes following adoptive transfer into lung recipients undergoing BOS pathogenesis. We found that CCR2^+^ monocyte differentiation into Mo-AMs was substantially dependent on TGF-β signaling during BOS pathogenesis. However, for iMacs, we observed comparatively less generation from WT monocytes, indicating TGF-β signaling may retard their development. Additionally, it is important to note that we could detect small numbers of Mo-AMs in tolerant allografts, indicating that Mo-AM generation is not sufficient to promote BOS. Interestingly, reports exist that clodronate-mediated TR-AM depletion prior to bleomycin treatment does not worsen pulmonary fibrosis (23). We also observed that clodronate-mediated donor TR-AM depletion and subsequent Mo-AM reconstitution does not spontaneously induce or increase the severity of BOS. Thus, our data point to the requirement for airway inflammation to trigger Mo-AM–dependent BOS development.

We found that PD-1 expression on intragraft CD8^+^ T cells largely marked the TRM cell compartment irrespective of tolerance status. Recent work in a mouse model of acute influenza infection has demonstrated that MHC class I, CD80, and CD86 are all required to maintain PD-1^+^ TRM cells (80). Interestingly, TRM cells in this setting were found to be exhausted, as PD-L1 blockade was required to clear secondary infection at the cost of developing fibrotic sequelae. In contrast, repeated PD-1 Ab blockade in a mouse kidney allograft model highly enriched for PD-1^+^ TRM cells failed to exacerbate chronic rejection (64). Similarly to our observations in the current study, these investigators did not find evidence of TRM cell exhaustion. Lung allograft TRM cells did not coexpress additional exhaustion markers and robustly recalled IFN-γ expression upon donor-antigen challenge by Mo-AMs. In contrast, TRM cell reactivation by TR-AMs was poor largely due to PD-L1 expression. These data therefore suggest that TR-AM may limit alloimmune responses. Alternatively, TR-AM could contribute to TRM cell development, as a recent report demonstrated that AM depletion prevents TRM cell differentiation in a murine influenza infection model (31). Overcoming PD-1–mediated inhibition of CD8^+^ T cell memory responses requires engagement of CD80 and CD86 (81), two costimulatory ligands expressed on both AM subsets. Our group has previously demonstrated that PD-1 expression on CD8^+^ T cells is required for costimulatory blockade–mediated induction of lung transplant acceptance as well as for prolonged interactions with recipient-derived intragraft CD11c^+^ cells (82). In this study, we detected prolonged interactions between allograft TRM cells and AMs that were donor-antigen dependent. Future intravital studies will be needed to assess whether TRM cell interactions with TR-AMs are also dependent on PD-1/PD-L1 engagement.

A notable feature of allografts with BOS is the accumulation of Gzmb^hi^Blimp-1^+^ TRM cells. Using an airway epithelial cell coculture system, we observed that BOS allograft TRM cells induced rapid and potent Gzmb-dependent proapoptotic activity. We also investigated the origins and requirements of the Gzmb^hi^Blimp-1^+^ TRM cell subset. Previous work in virally infected mice has demonstrated that skin TRM cells can maintain themselves locally from a pool of preexisting TRM cells (67, 83). Our finding that Blimp-1^+^Gzmb^hi^ TRM cells can be generated from TRM cells isolated from tolerant allografts is in line with these previous reports and suggests that specifically targeting intragraft TRM cells may be a viable strategy for preventing BOS. However, our studies do not rule out a possible contribution from recruited peripheral TRM cell precursors. Insight into the in vivo antigen-presenting cell requirements for Blimp-1^+^Gzmb^hi^ TRM cell generation was gained by observations of sharply lower numbers of these cells in TGF-βR2^Δ/Δ^ recipients, which during BOS pathogenesis, could produce iMacs and CD11b^+^ DCs, but not Mo-AMs. When these data are considered in conjunction with the ability of Mo-AMs to induce TRM cell IFN-γ expression, our results support the notion that Mo-AM–mediated antigen presentation is a key promoter of TRM cell activation and expansion.

Previous reports differ as to whether Blimp-1 expression is required for TRM cell generation. One reason for this possibility is the existence of tissue-specific transcriptional programs for TRM cell development or maintenance. For example, Mackay and colleagues demonstrated that Blimp-1 is required for the bulk of liver but not skin TRM cell development (84). Later work by Behr and colleagues examined Blimp-1 requirements for lung TRM cell formation and found that it was critical for Gzmb expression and CD103^+^CD69^+^ TRM cells, but not CD103^–^CD69^+^ TRM cell development (85). Although this group did not report Blimp-1 requirements for CD49a expression, in line with their observations, we found Blimp-1 was necessary for TRM cell Gzmb, but not CD69 expression in allograft TRM cells. Additionally, we only observed mild CD103 TRM cell expression that was specific for allografts with BOS. The reasons for this are not clear, but it is presumably due to the need for high TGF-β levels for CD103 expression (86). CD49a and CD69 play well-established roles in TRM cell survival, trafficking, and retention, while the role of CD103 on the lung TRM cells is less understood, although it has been suggested to promote epithelial adherence (87). In particular, recent work has demonstrated that CD49a is a common marker of TRM cell cytolytic activity irrespective of CD103 expression (88). In line with these reports, we observed that Blimp-1^hi^ TRM cells from lung allografts with BOS expressed high levels of CD49a and Grzmb readily killed pulmonary epithelial cells. Finally, we noted that Blimp-1^Δ/Δ^ allograft recipients generated high numbers of Mo-AMs, providing further evidence that this AM subset drives BOS through promoting Blimp-1–dependent TRM cell expansion.

There are several limitations to this study. These include that our mouse BOS lung transplant model also shows some diffuse parenchymal damage, possibly due to CCSP expression in bronchioalveolar stem cells (89, 90). Also, we cannot completely eliminate the possibility that other CCR2^+^-expressing cells may contribute to BOS pathogenesis (76). Nevertheless, we directly observed the ability of CCR2^+^ monocytes to generate lung allograft Mo-AMs, which in turn promoted the expansion and activation of TRM cells, leading to BOS. Finally, because TGF-β signaling also plays an important role in AM maintenance (25), it was not possible to clearly distinguish between the relative contribution of CCR2^+^ monocyte recruitment, differentiation, and Mo-AM maintenance on overall Mo-AM intragraft accumulation. Future studies involving dissection of downstream components of the TGF-β–signaling pathway could allow for better discrimination between the various mechanisms that drive Mo-AM accumulation as well as an understanding of their impact on chronic rejection. In conclusion, we have identified an inducible functional plasticity within the AM compartment that can be harnessed to lower TGF-β bioavailability and prevent BOS. Our data also extend the notion that AMs can be programmed to alter adaptive immune responses with implications beyond the transplantation field.

## Methods

### Mice and orthotopic lung transplantation.

FVB, C57BL/6 (B6), B6. Thy1.1, B6. Lyz^Cre^, B6. CD8^Cre^, B6. Prdm1^fl/fl^, B6. Tgfbr2^fl/fl^, and B6. β-actin EGFP mice were all purchased from Jackson Laboratory. CCR2^DTR^, Dcn^fl/fl^, and CCR2^CreERT2^ mice were gifts from Eric G. Pamer (University of Chicago, Chicago, Illinois, USA), David E. Birk (University of South Florida, Tampa, Florida, USA), and Burkhard Becher (University of Zurich, Zurich, Switzerland), respectively. 2T-FVB and 3T-FVB donor mice and mouse left orthotopic lung transplantation procedures have been previously described by our group (6). To induce allograft acceptance, recipients received i.p. 250 μg of CD40L Abs (clone MR1) on POD0 and 200 μg of mouse recombinant CTLA4 Ig on POD2 (74). Club cell injury was triggered by DOX ingestion via food (625 mg/kg chow; ENVIGO) and water (2 mg/ml, MilliporeSigma) for 2 to 2.5 days. Tamoxifen (MilliporeSigma) was dissolved in Mazola corn oil and injected i.p. 5 times at 0.25 mg/g body weight every other day, and then mice were rested 5 days prior to DOX ingestion. Diphtheria toxin (Sigma-Millipore) was dissolved in PBS and injected i.p. 1 day prior to DOX ingestion at 10 ng/g body weight.

### ECP.

B6 (syngeneic) leukocytes were isolated from splenocytes by centrifugation at 400*g* through a density separation medium (Lympholyte-M, Cedarlane) to eliminate dead cells, debris, and erythrocytes. The remaining erythrocytes were removed by ACK lysing buffer, and leukocytes were resuspended at 5 × 10^6^ cells/ml in complete medium with 8-MOP (200 ng/mL), incubated in the dark for 30 minutes at 25°C, and irradiated at 2 J/cm^2^ UVA in an ECP irradiator box (Johnson & Johnson). Following DOX ingestion, recipients received 3 i.v. doses of 10^7^ ECP-treated leukocytes in 100 μL normal saline spaced at 3-day intervals.

### Immunohistological staining and collagen analysis.

Harvested grafts were formaldehyde fixed, paraffin embedded, and stained with H&E or Masson’s trichrome stain. Lung transplant histology was graded by a blinded pathologist using the 2007 revision of the 1996 working formulation for the standardization of nomenclature in the diagnosis of lung rejection (35). For immunohistochemical analysis, paraffin sections were first blocked with 5% goat serum and 2% fish gelatin (both from Sigma-Aldrich) at 25°C for 45 minutes. Sections were then stained with 1:500 polyclonal rabbit anti-mouse/rat CCSP (catalog WRAB-3950, Seven Hills Bioreagents), 1:100 monoclonal rabbit anti-mouse CD64 (clone MA5-29706, Thermo Fisher), 1:100 polyclonal goat anti-mouse DCN (catalog AF1060, R&D Systems), and mouse anti-acetylated tubulin, 1:5,000 (clone 6-11B-1, Sigma-Aldrich) overnight at 4°C. Mouse anti-human CD8α (clone C8/114B), rat anti-human (clone 16G6), and rabbit anti-human polyclonal CD49a (catalog PA5-95563) were all diluted at 1:200 (Thermo Fisher). For secondary Ab-mediated immunofluorescent visualization, we used 1:1,000 goat anti-mouse Alexa Fluor 488–labeled secondary Abs (catalog A-11-001, Thermo Fisher), 1:1,000 donkey anti-goat Alexa Fluor 488 (catalog A-11055, Thermo Fisher), 1:1,000 donkey anti-rabbit Alexa Fluor 555 (catalog A-31572, Thermo Fisher), and 1:1,000 goat anti-rabbit Alexa Fluor 555 (catalog 4413S, Cell Signaling Technology). For collagen measurements, 10 mg of allograft tissue was analyzed with a Hydroxyproline Assay Kit (MilliporeSigma) in accordance with the manufacturer’s recommendations.

### Flow cytometric analysis and antigen recall assays.

Lung tissue was minced and digested in an RPMI 1640 solution with type 2 collagenase (0.5 mg/mL) (Worthington Biochemical) and 5 units/mL DNAse (MilliporeSigma) for 90 minutes at 37°C and then filtered through a 70 μm cell strainer (Thermo Fisher) and treated with ACK lysing buffer (Worthington Biochemical). Live cell discrimination was conducted with Zombie (BioLegend) Fixable Dye. Cell surface staining was conducted with the following Abs: CD45 (clone 30-F11; eBioscience), CD45.2 (clone 104; BioLegend), CD90.2 (clone 53-2.1; eBioscience), CD4 (clone RM4-5; eBioscience), CD8α (clone 53-6.7; eBioscience), CD31 (clone 390; BioLegend), CD34 (clone HM34; BioLegend), and CD326 (clone G8.8; BioLegend). Staining for Foxp3 (FJK-16s, eBioscience), Ki-67 (16A8; BioLegend), and CCSP (Seven Hills Bioreagents) was conducted with the Intranuclear Transcription Factor Staining Buffer Kit (Invitrogen) in accordance with the manufacturer’s recommendations. For IFN-γ and IL-17A expression, cells were first stimulated with 1 μM ionomycin (MilliporeSigma) and 20 ng/ml PMA (MilliporeSigma) for 3.5 hours, with 2 μM Golgi Plug (BD Biosciences) added for the last 3 hours of stimulation. Cells were then stained with IFN-γ (clone XMG1.2; eBioscience) and IL-17A (clone TC11-18H10.1; BioLegend) using a Cytofix/Cytoperm kit (BD Biosciences) in accordance with the manufacturer’s recommendations. For antigen-specificity measurements, T cells were fractionated by positive selection using CD4^+^ or CD8^+^ immunomagnetic beads (Miltenyi Biotec) from allograft cell suspensions and cocultured at a 3:1 ratio with irradiated T cell–depleted FVB or B6 cell splenocytes for 96 hours and pulsed with 0.5 μg/ml K–α1 tubulin and Col V (obtained from T. Mohannakumar, St. Joseph’s Hospital, Phoenix, Arizona, USA). IFN-γ and IL-17A were measured with uncoated ELISA kits from Invitrogen in accordance with the manufacturer’s recommendations.

### Semi-quantitative RT-PCR.

FACS-sorted AMs were extracted for RNA with RNAeasy kits (QIAGEN) and reverse transcribed with a high-capacity cDNA reverse transcription kit in accordance with the manufacturer’s instructions (Thermo Fisher). TaqMan Gene Expression Assays specific for indicated genes (Thermo Fisher) were used to assess transcript levels following normalization against the macrophage housekeeping gene *Stx5a* (91).

### HA analysis.

Low-endotoxin HA was purchased from MilliporeSigma. HA quantitation within BALF was conducted by sandwich ELISA (Echelon Biosciences) in accordance with the manufacturer’s recommendations.

### TGF-β measurements.

TGF-β isoforms were measured with Bio-Plex Pro TGF-β Assays (Bio-Rad) in accordance with the manufacturer’s directions. To measure TGF-β activity, the NIH/3T3 SMAD2/3-luciferase reporter cell line (Signosis) was cocultured with BALF and extracted with RPMI 1640 (3:1 v/v ratio) or platelet-free plasma at a 1:10 (v/v) ratio for 16 hours. Luciferase activity was measured using a BioTek Synergy/HTX Multi-Mode Reader.

### Cell culture.

FACS-sorted CD45.2 Mo-AMs and CD45.1^+^ TR-AMs from either 3T- or 2T-FVB allograft recipients or B6 mice were seeded into 96-well round-bottom plates at 5.0 to 7.5 × 10^4^ per well and cocultured with PD-1^+^CD49a^+^CD8^+^ T cells FACS-sorted from either 2T- or 3T-FVB allograft recipients at a 1:1 ratio in the presence of 10 μg/ml anti-mouse PD-L1 Abs (clone BE0361; Bio X Cell) or control rat IgG for 72 hours. Cultures were stimulated with 20 ng/ml PMA for 3 hours and assessed with a mouse IFN-γ ELISA kit (MilliporeSigma). For airway epithelial cell culture, FVB lung tissue cell isolates were prepared as described for FACS preparation and incubated with biotin-conjugated Abs specific for CD45.1 (clone A20), CD34 (clone RAM34), CD31 (clone MEC13.3), CD90.1 (clone HIS51), and CD15 (clone mc-480) (all from eBioscience), washed, and then labeled with anti-biotin MicroBeads (Miltenyi Biotec) for negative selection on LS columns (Miltenyi Biotec). The remaining cells were then incubated with biotin-conjugated CD326 Abs (clone caa7-9G8, Miltenyi Biotec), washed, and then labeled with anti-biotin MicroBeads for MS column–mediated (Miltenyi Biotec) positive selection. Enriched club cell fractions were resuspended in MTEC/Plus Medium and seeded at 3.0 ×10^4^ cell per well in flat-bottom 96-well tissue culture plates (Thermo Fisher) coated with 50 μg/ml type I rat tail collagen (BD). 3T-FVB allograft TRM cells were added to epithelial cells at a 1:1 ratio, and MitoTracker DeepRed FM and MitoSOX (Both from Thermo Fisher) were added at 1 μM to cultures 30 minutes prior to removal for FACS analysis. DNA fragmentation was measured using the TUNEL FACS-Based Assay Kit (Abcam) according to the manufacturer’s recommendation.

### PET imaging.

A 0- to 60-minute dynamic PET/CT scan was performed following injection of ^64^Cu-DOTA-ECL1i (100 μCi in 100 μL saline) using the Inveon PET/CT System (Siemens). The PET images were reconstructed with the maximum a posteriori algorithm and analyzed by Inveon Research Workplace. Organ uptake was calculated as percentage of injected dose per gram (%ID/g) of tissue in 3D regions of interest without correction for partial volume effect. Reagents, synthesis, and characterization of all compounds have been previously described by our group (92).

### 2-Photon intravital imaging.

Chest wall exposure was conducted between the third and seventh ribs, and a cover glass slide was adhered to the lung allograft using tissue glue (VetBond) applied in a gentle manner as not to disturb blood flow. AMs were imaged with PE-Siglec F (2 μg, clone S17007L; BioLegend) administered i.v. 30 minutes after engraftment. FACS-sorted PD-1^+^CD49a^+^CD69^+^CD8^+^ T cells isolated from e3T-FVB allografts were labeled with 5 μM CFSE, and between 1 and 3 × 10^5^ cells were intratracheally administered 1 day before imaging. Data were collected by sequential z sections (24, 2.5 μm each), which were acquired in an imaging volume of 200 × 225 × 60 μm^3^. Analyses were performed with Imaris (Bitplane). Associations between AMs and TRM cells were defined as physical interactions that lasted longer than 15 seconds. For each lung transplant, at least 5 areas were examined up to approximately 50 μm deep. Data shown has been pooled from at least 3 mice per group.

### Statistics.

Data were analyzed by the Mann-Whitney *U* test or 1-way ANOVA and are represented as mean ± SD. Statistical analysis was conducted with GraphPad Prism software, version 9.0. *P* < 0.05 was considered significant.

### Study approval.

Animal experiments were conducted in accordance with an approved IACUC protocol (Washington University, 19-0827). Human lung transplant tissue was obtained in accordance with an approved IRB (Washington University, 201012829).

## Author contributions

The investigation was conducted by ZL, FL, JZ, DZ, GSH, HPL, DS, AP, MC, WL, and AEG. RH, DEB, LKT, HJH, ASK, BWW, HSK, and AEG contributed to methodology. HJH, YL, and AEG conceived the project. YL, DK, and AEG wrote the manuscript.

## Supplementary Material

Supplemental data

Supplemental video 1

Supplemental video 2

## Version 1 10/03/2022

Electronic publication

## Version 2 10/04/2022

Howard J. Huang's name corrected in author list
