# Supplementary material for: Reprogramming alveolar macrophage responses to TGF-β reveals CCR2+ monocyte activity that promotes bronchiolitis obliterans syndrome
Source: J Clin Invest. 2022 Oct 3;132(19):e159229. doi: 10.1172/JCI159229 (PMC9525120; doi:10.1172/JCI159229)
Supplement: Supplemental data [file jci-132-159229-s010.pdf]

Figure S1

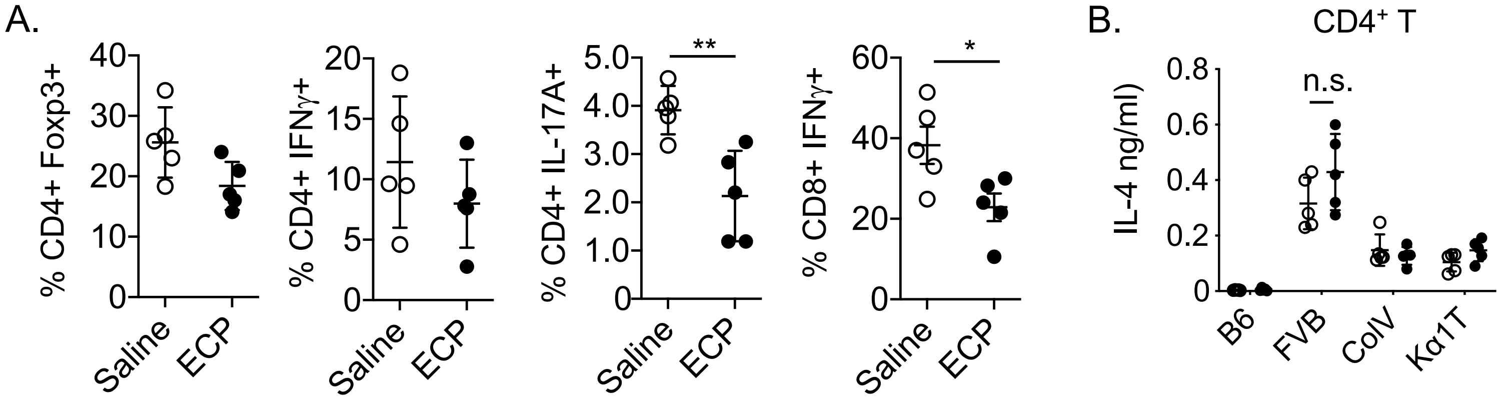

Fig. S1: **ECP effects on T cell activation**. (**A**) Intragraft abundance for indicated effector T cells. Data shown is mean percent abundance ± S.D.; 2-sided Mann Whitney U test; *p< 0.05, **p < 0.01. (**B**) IL-4 expression following stimulation of saline (open circles) or ECP-treated (closed circles) intragraft $CD4^+$ T cells with B6 splenocytes (syngeneic antigens), FVB splenocytes (donor antigens) or B6 splenocytes pulsed with lung self-antigens Col V and Kα1T (N=5/group). Data shown are mean IL-4 culture supernatant concentration ± S.D.; 2-sided Mann Whitney U test; where n.s. means non-significant.

Figure S2

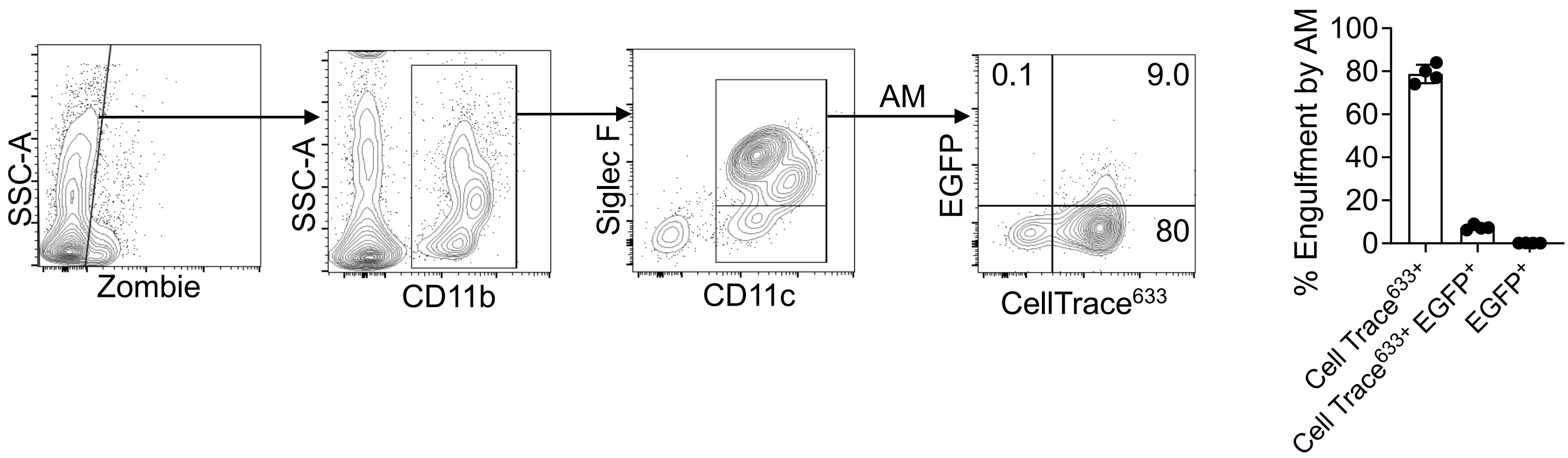

Fig. S2: **ECP-treated leukocytes are rapidly engulfed by AM**. Cell Trace^{633}-labeled ECP-treated leukocytes and control EGFP^{+} non-ECP-treated leukocytes were co-transferred into 3T-FVB lung recipients and AM engulfment was analyzed two hours later. FACS plots are a representative result from 4 experiments. Histograms show pooled data of 4 experiments with mean AM percent engulfment ± S.D.

Figure S3

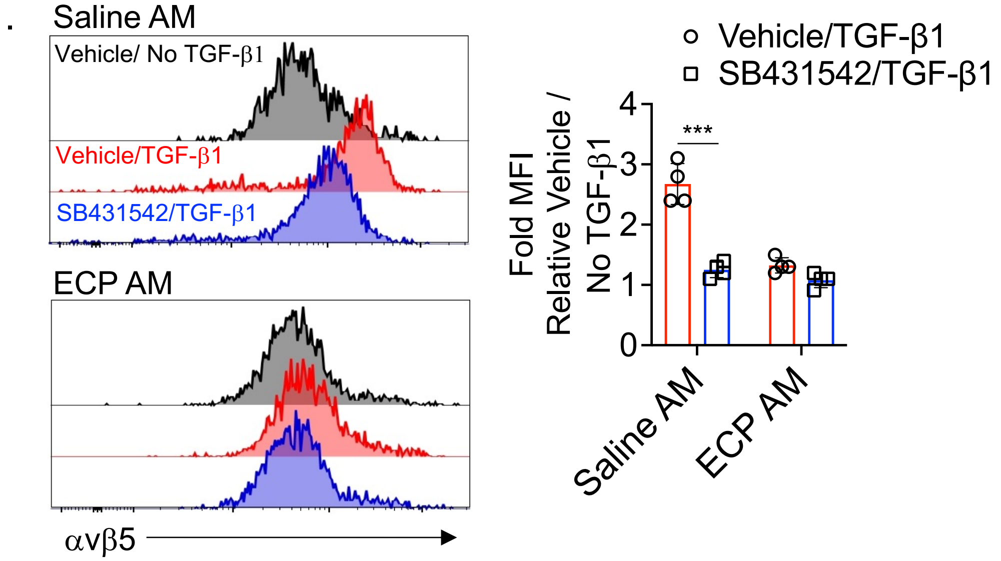

Fig. S3: **ECP-treated leukocytes are engulfed by AM, which in turn induces resistance to TGF-β mediated αvβ5 upregulation**. Saline and ECP-treated AM were pre-treated with 10 mM SB43152 or vehicle (DMSO) and then stimulated with or without TGF-β1. FACS plots shown are a representative result from 4 experiments. Fold MFI changes in αvβ5 expression is normalized to respective non-TGF-β-treated DMSO-pretreated controls. Data in bar graphs is pooled data from 4 experiments; Mean Fold MFI ± S.D.; 2-sided Mann Whitney U test; where ***$p < 0.001$.

Figure S4

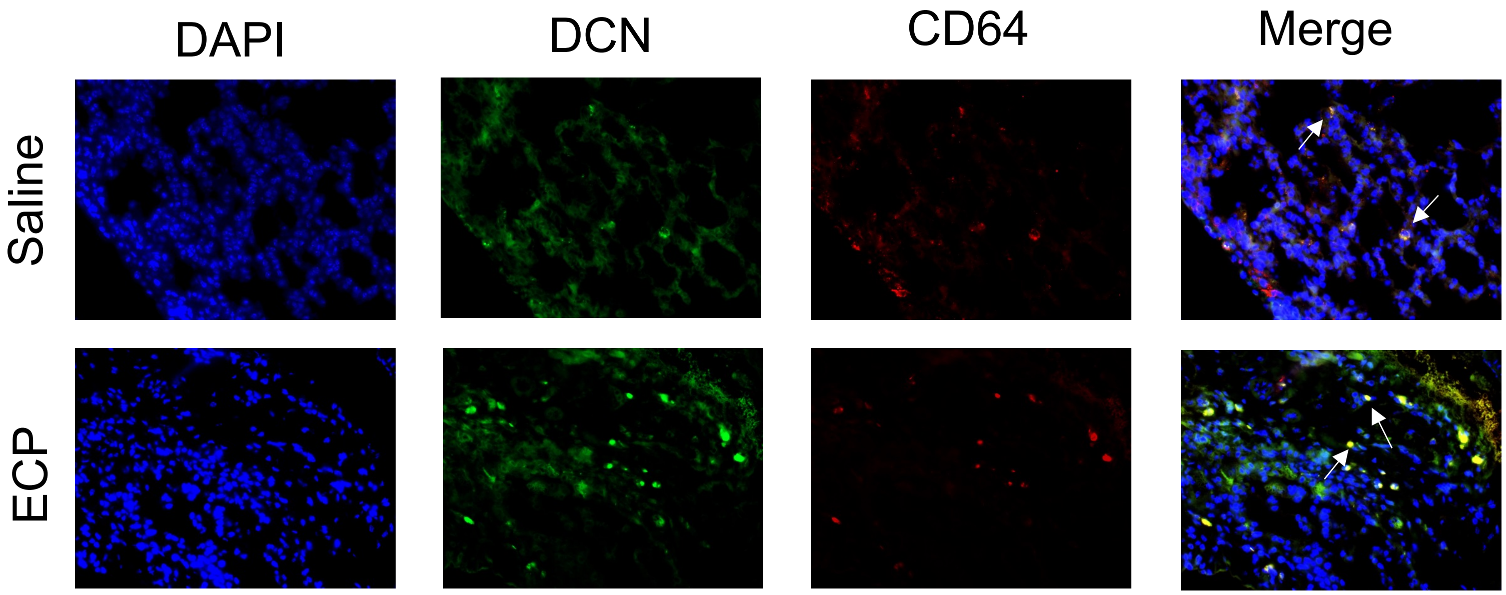

Fig. S4: **ECP stimulates DCM expression in lung allograft CD64+ macrophages following induction of BOS pathogenesis**. POD 16 3T-FVB allograft tissue treated with saline vehicle (N=3) or ECP (N=4) and analyzed for CD64 and DCN expression. Arrows show $CD64^{+}$ DCN^{+} macrophages in alveolar spaces. Data shown are a representative result for each group.

Figure S5

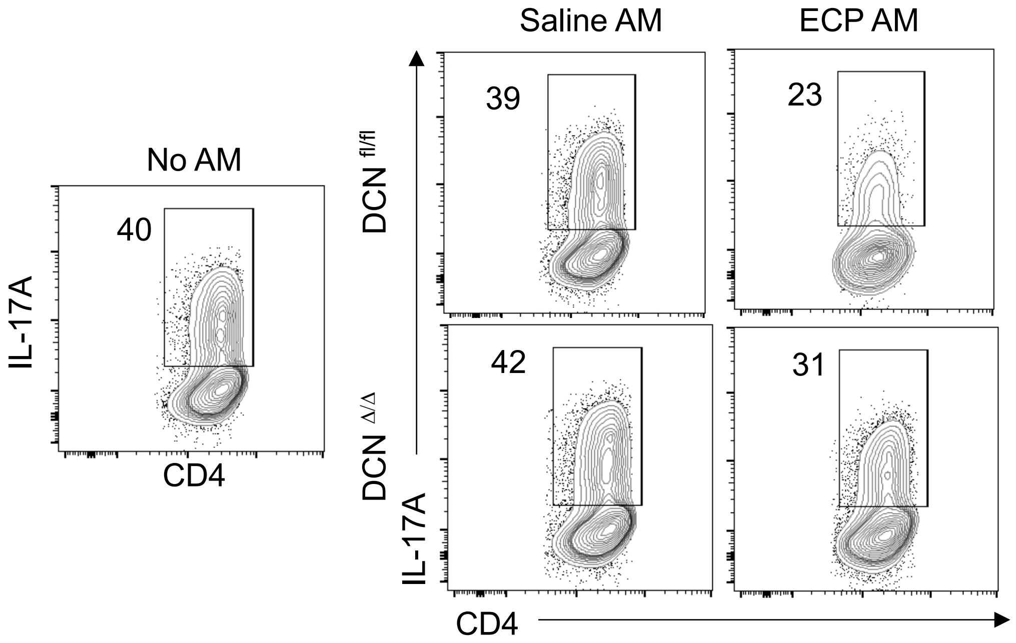

Fig. S5: **ECP-treated AM inhibits T_h17 development in DCN expression-dependent manner.** A representative FACS plot result from 5 experiments where plate bound CD3ε and CD28 Abs were used to activate naïve B6 $CD4^+$ T cells in the presence or absence of indicated AM conditioned supernatants added in a 1:1 v/v ratio to T_h17 polarization medium containing 10 ng/ml TGF-β1. Intracellular IL-17A expression was assessed 4 days later.

Figure S6

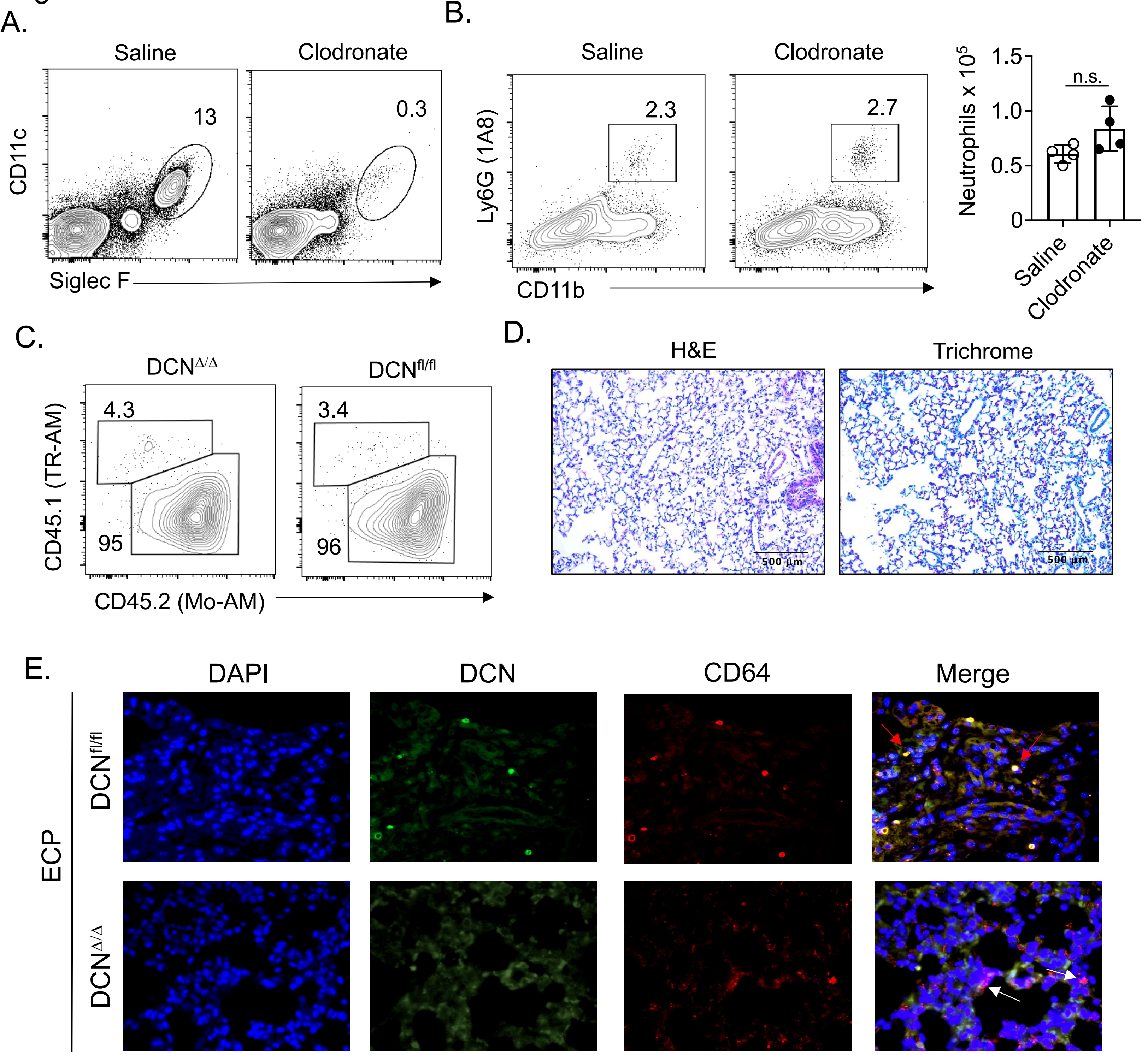

Fig. S6: **Reconstitution with recipient-derived AM does not spontaneously induce OB lesions and validation of DCN antibody specificity. (A)** AM depletion of donor lungs one day after clodronate depletion. Data shown is a representative FACS plot result from N=2/group (**B**) Intragraft neutrophilia in donor lungs one day after receiving clodronate liposomes. Data shown are representative FACS plots from N=4/group where histograms depict mean neutrophil numbers ± S.D; 2-sided Mann Whitney U test; n.s. is non-significant. **(C)** Mo-AM generation in clodronate liposome-treated POD 7 $DCN^{\Delta/\Delta}$ and $DCN^{fl/fl}$ lung recipients of FVB lungs. Data shown are representative FACS plots from N=2/group. (**D)** POD 7 histology of a 3T-FVB allograft from a $DCN^{\Delta/\Delta}$ recipient that received clodronate liposome treatment. Histology shown is a representative result from 2 transplants. (**E**) POD 16 allograft tissue from ECP-treated $DCN^{\Delta/\Delta}$ and $DCN^{fl/fl}$ recipients of 3T FVB lungs evaluated for DCN expression within the CD64 compartment. Red arrows show CD64+ macrophages that express DCN. White arrows show CD64+ macrophages that lack DCN expression. Data shown are representative allograft stains from N=3/group.

Figure S7

A.

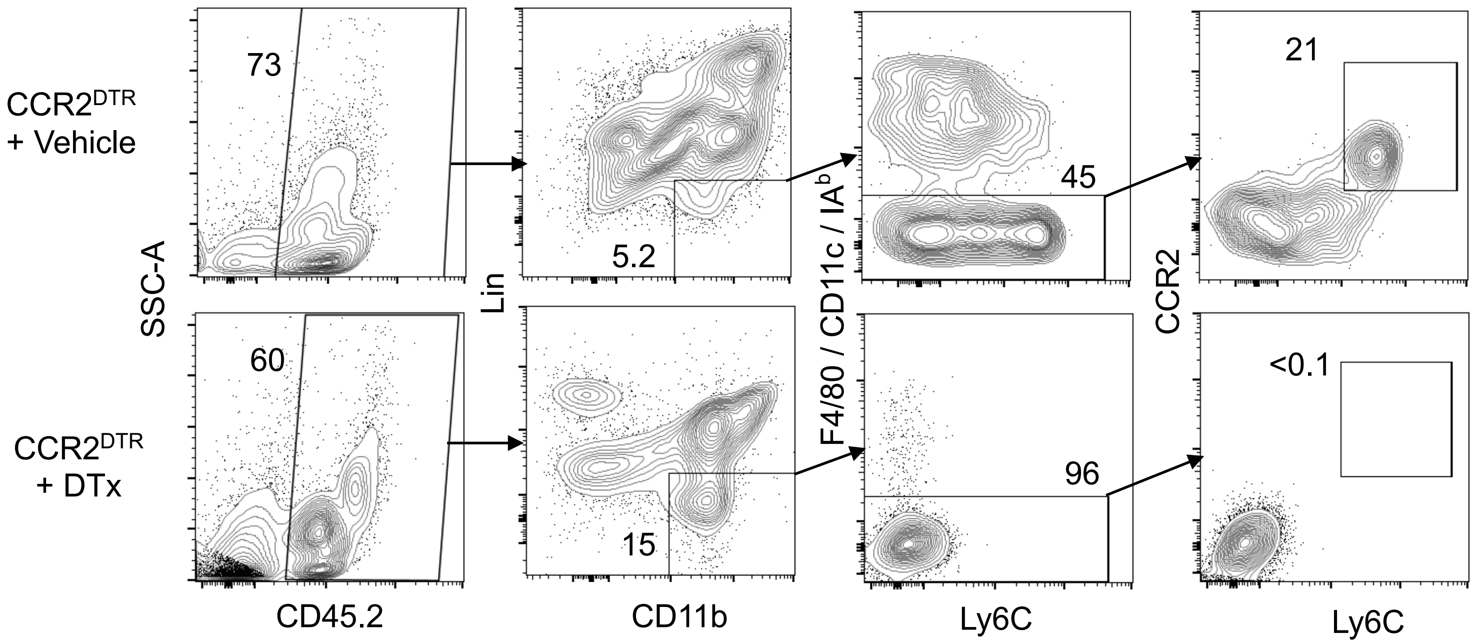

B.

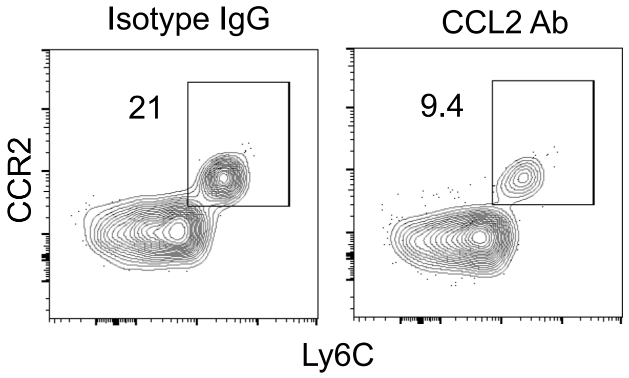

Fig. S7. Validation of monocyte depletion within lung recipients. **(A)** FACS plot gating strategy to measure allograft CCR2^{+} monocyte abundance of CCR2^{DTR} recipients of 3T FVB allografts 3 days after receiving PBS or 10 ng/g body weight of i.v. diptheria toxin (DTx). Data shown is a representative result of 2 independent experiments where Lin represents a cocktail of CD90.2, B220, NK1.1 and Ly6G-specific Abs. **(B)** B6 recipients of 3T FVB allografts received 200 μg i.v. of CCL2 neutralizing Abs on POD 6, 9 and 12 and assessed for blood peripheral CCR2^{+} accumulation on POD 16. FACS plots shown is representative result from 2 experiments.

Figure S8

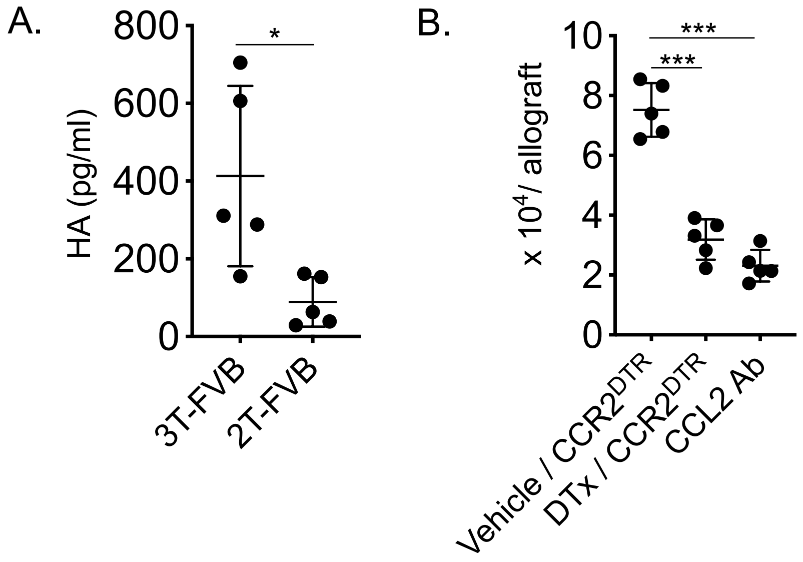

Fig. S8. HA accumulation in 3T-FVB allografts and CCR2 activity inhibition prevents intragraft Mo-AM accumulation. (A) HA concentration in the BAL fluid from POD 16 3T-FVB and 2T-FVB allograft. Data shown is a representative result from 2 determinations for N=5/group with a mean HA concentration ± S.D.; 2-sided Mann Whitney U test; *$p<0.05$. Intragraft 3T FVB Mo-AM numbers transplanted into POD16 $CCR2^{DTR}$ recipients that received indicated treatments or transplanted into B6 recipients that received CCL2 Ab. Data shown is for N=5/group with mean Mo-AM numbers ± S.D; 1-way ANOVA with Dunnett's multiple comparisons test; ***$p<0.001$.

Figure S9

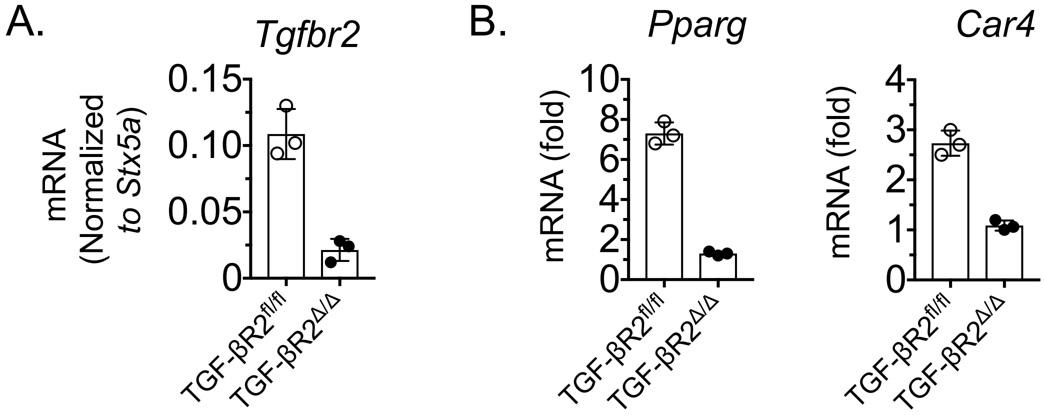

Fig. S9. **Validation of TGF-βR2 deletion in CCR2^{+} monocytes**. TGF-βR2$^{fl/fl}$ and TGF-βR2$^{\Delta/\Delta}$ mice were treated tamoxifen i.p. every other day for 10 days, rested for 5 days, FACS sorted for peripheral CCR2^{+} blood monocytes and assessed for (**A**) *Tgfbr2* transcript levels or (**B**) stimulated overnight with 5 ng/ml of TGF-β1 and measured for *Pparg* and *Car4* transcript levels. For **(A)** data shown is normalized to the macrophage housekeeping gene *Stx5a* and (**B**) shown normalized to TGF-βR2$^{\Delta/\Delta}$ levels where bars represent means ± S.D. Data shown for **A** and **B** are representative results from 2 determinations.

Figure S10

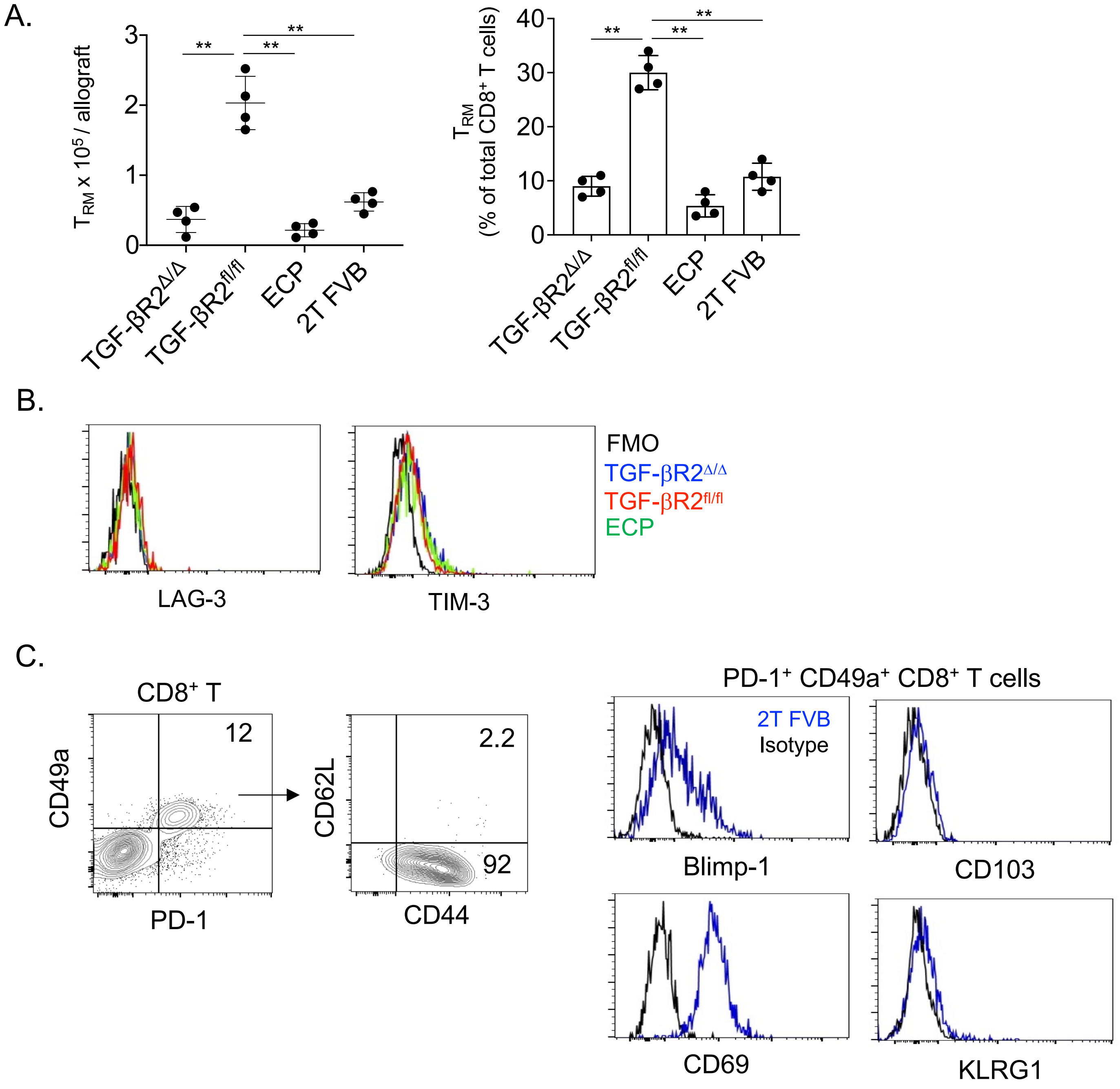

Fig. S10. **T_{RM} phenotype and abundance in lung transplants.** **(A) I**ntragraft numbers and percent abundance of T_{RM} for indicated transplant and treatment conditions. Bars represent mean levels ± S.D.;N=4/group; 1-way ANOVA with Dunnett's's multiple comparisons test **p < 0.01. (**B**) A representative FACS histogram result of T_{RM} LAG-3 and TIM-3 expression of N=4/group. (**C**) 2T-FVB allograft T_{RM} marker expression patterns. Data shown are a representative result from N=4/group.

Figure S11

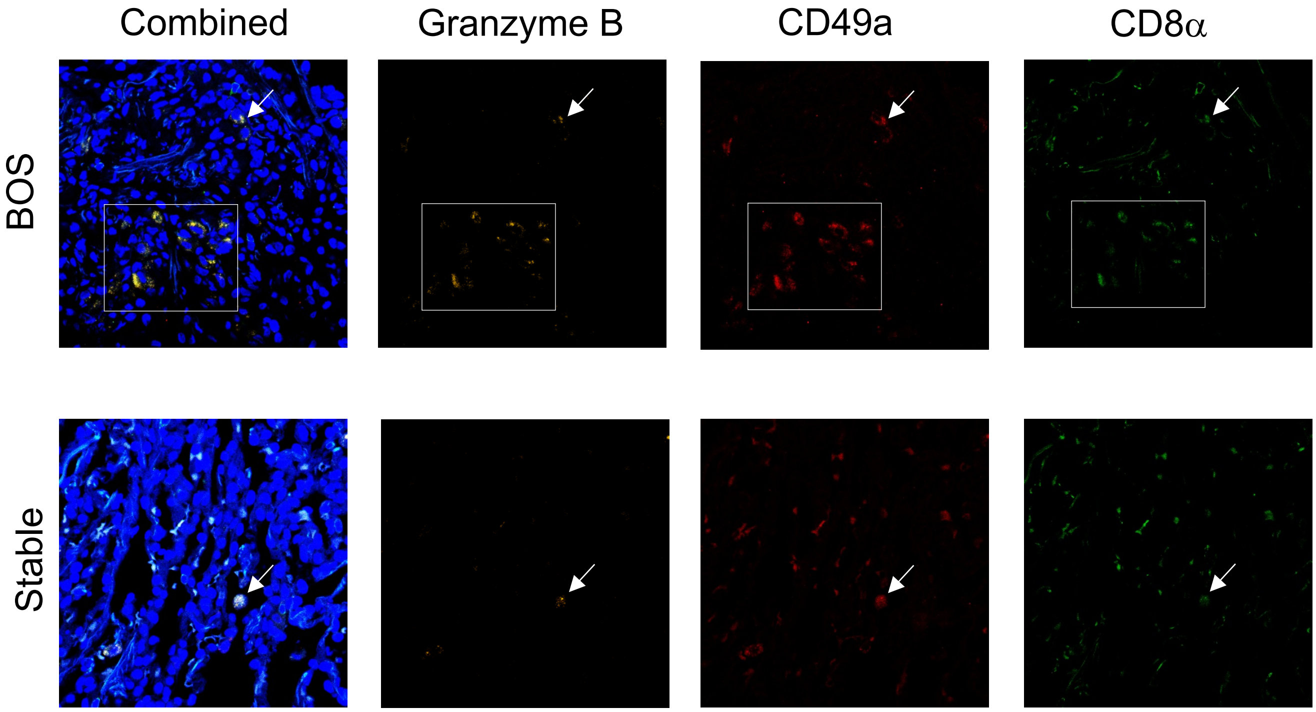

Fig. S11. Clusters of Gzmb⁺CD49a⁺CD8⁺ T cells in BOS subjects. Explant tissue from patients with BOS or core biopsies from patients without evidence of CLAD or antibody-mediated rejection (Stable) were stained with indicated antibodies. Images shown are representative stains from BOS (N=4) and stable recipients (N=6). The white box represents a cell cluster and arrows show a single cell.

Figure S12

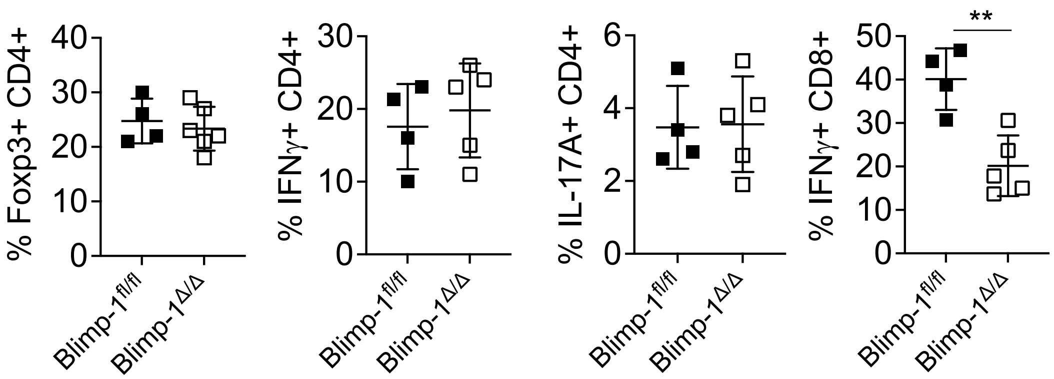

Fig. S12. **CD8$^+$ T cell-specific Blimp-1 deficiency reduces intragraft IFN-γ^+ CD8$^+$ T cell accumulation.** Flow cytometric analysis of indicated intragraft effector T cells in Blimp-1^{flfl} and Blimp-1$^{\Delta/\Delta}$ recipients (N=4/group). Data shown is mean percent abundance ± S.D.; 2-sided Mann-Whitney U test; **p < 0.01.
